# Cross‐Platform Biosensing of Immune Receptors Using Peptide‐Functionalized Graphene

**DOI:** 10.1002/advs.202519436

**Published:** 2025-12-12

**Authors:** Ahmar Hasnain, Heiko Heilmann, Annabel Pohl, Celine Weber, Bernd Bufe, Alexey Tarasov

**Affiliations:** ^1^ Nanoelectronics & Biosensing Lab Faculty of Computer Sciences and Microsystems Technology Kaiserslautern University of Applied Sciences Amerikastr. 1 66482 Zweibrücken Germany; ^2^ Molecular Immunology Lab Faculty of Computer Sciences and Microsystems Technology Kaiserslautern University of Applied Sciences Amerikastr. 1 66482 Zweibrücken Germany

**Keywords:** biosensing, graphene field‐effect transistor (gFET), immune receptors, peptide‐functionalized graphene, surface plasmon resonance (SPR)

## Abstract

Changes in the composition of specific cell‐surface molecules on immune cells are important early markers of directed immune responses. Current biosensors largely focus on detecting soluble disease markers. Here, a peptide‐functionalized graphene biosensor is presented capable of platform‐independent, highly sensitive detection of specific immune receptors on living cells. Formyl peptide receptor 2 (FPR2) is targeted, a key modulator of innate immune cells, and validated the technology in two complementary formats: i) graphene‐enhanced surface plasmon resonance (SPR) as a robust optical benchmark, and ii) graphene field‐effect transistors (gFETs) as a compact, cost‐effective electrical alternative. Target specificity is first confirmed using HEK293T cells selectively overexpressing FPR2. Detection of FPR2 on primary human neutrophils is achieved with high reproducibility using fewer than 10 000 cells mL^−1^, demonstrating both sensitivity and reliability. By demonstrating sensitive and reproducible detection across both optical and electrical platforms, this work bridges materials science and immunology, highlighting the potential of peptide‐functionalized graphene biosensors for point‐of‐care diagnostics, immune monitoring, and early sepsis triage.

## Graphical abstract

A peptide‐functionalized graphene biosensor enables sensitive and selective detection of living immune cells. Targeting Formyl‐Peptide Receptor 2 (FPR2), the assay is demonstrated on model cells and primary human neutrophils. The same assay is applied to two complementary platforms: graphene‐enhanced surface plasmon resonance for optical benchmarking and graphene field‐effect transistors for compact electrical readout, highlighting potential for immune monitoring and point‐of‐care diagnostics.

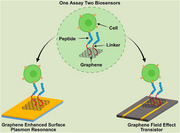

## 1 Introduction

Immune cells express many different kinds of surface receptors that can recognize particular molecular signatures of pathogens or patterns of tissue damage. Their activation alerts and recruits immune cells.^[^ ^1^, ^2^, ^3^ ^]^ Alterations in the composition of cell surface receptors can be used as an early marker for different diseases.^[^ ^4^, ^5^ ^]^ Therefore, biosensor technologies that permit the specific detection of such molecules may be of great benefit because they can support and advance immune receptor research.^[^ ^6^, ^7^ ^]^ Moreover, they can increase the limited number of disease biomarkers, which are currently majority demanded to be disease‐specific, soluble proteins that are easy to monitor.^[^ ^8^ ^]^ Furthermore, many of these cell surface receptors are of high relevance for ongoing research and yet there is still a lot to be learned about their physiological roles in diseases and host defence.^[^ ^1^, ^5^ ^]^ Thus, biosensors that are able to address specific cell surface molecules on living immune cells could also provide many new opportunities in this field. However, the requirement of large quantities of biological starting material as well as being dependent on expensive readout‐platforms can complicate the design of suitable biosensors.^[^ ^9^, ^10^, ^11^ ^]^

Monolayer graphene‐based biosensors may be a promising route to facilitate the detection of specific surface receptors on blood immune cells and to meet the challenges related to this task.^[^ ^11^, ^12^ ^]^ Their label‐ and amplification‐free detection may allow sensitive quantification of target receptors. Moreover, chemical vapor deposition (CVD)‐grown monolayer graphene offers excellent optical and electrical properties that are suitable for point‐of‐care (POC) devices.^[^ ^12^, ^13^ ^]^ Graphene field‐effect transistors (gFETs) are particularly attractive because they are compact, cost‐effective, and portable, making them strong candidates for translational diagnostics^[^ ^6^, ^14^, ^15^, ^16^ ^]^ and monitoring of treatment responses.^[^ ^17^ ^]^ In contrast, surface plasmon resonance (SPR) represents a more established platform that is highly suited for assay development and benchmarking.^[^ ^12^, ^18^, ^19^ ^]^ Incorporating a single graphene monolayer onto an SPR chip can substantially enhance sensitivity, selectivity, and reproducibility, while also enabling real‐time monitoring of binding events.^[^ ^12^ ^]^ Multichannel SPR further allows parallel testing of capture ligands against controls on the same chip, providing a rigorous framework for optimizing receptor‐ligand assays.^[^ ^20^ ^]^ Once validated with SPR, these sensing principles can be translated into gFETs, where graphene's large surface‐to‐volume ratio, biocompatibility, and cell‐supporting properties facilitate robust and portable detection of immune cells.^[^ ^11^, ^21^ ^]^ Integrating graphene's optical enhancement with scalable electronic transduction creates a versatile, dual mode biosensing strategy. In this framework, SPR serves as the benchmark platform for developing and validating receptor‐specific assays, while gFETs represent the translational target due to their portability, scalability, and cost‐effectiveness. By enabling optical and electrical readouts on the same peptide‐functionalized surface, our approach allows direct comparison of modalities without altering the biology.

To establish this type of technology, we chose formyl peptide receptors (FPRs) that are expressed on innate immune cells where they regulate inflammation by recognizing pathogen‐derived or endogenous ligands in the context of many diseases such as bacterial infection, Alzheimer's disease or cancer, as a relevant immunological target.^[^ ^4^, ^22^, ^23^, ^24^, ^25^, ^26^, ^27^, ^28^ ^]^ In detail, we focused on the FPR2 subtype, and because of this receptor's capability to detect small peptides, we were able to select WKYMVm‐NH2 as a target peptide^[^ ^4^, ^28^, ^29^, ^30^ ^]^ and f‐MEQQNK as a non‐interacting control. To validate peptide‐receptor specificity, we first employed HEK293T cells, a human embryonic kidney cell line engineered to transiently overexpress FPR2. This model confirmed that the peptide‐functionalized graphene interface could reliably capture FPR2‐expressing cells in both SPR and gFET formats with high sensitivity and reproducibility. Having established the assay, we then demonstrated its transferability to primary human neutrophils, which naturally express high levels of FPR2.^[^ ^31^ ^]^ Importantly, neutrophil detection was robust, specific, and achievable with low cell numbers in both modalities. Given the role of FPRs in immune responses and their dysregulation in disease,^[^ ^32^, ^33^ ^]^ this peptide‐graphene biosensor platform offers significant potential for early disease diagnosis, immune monitoring, and rapid assessment of inflammatory states such as sepsis. The combination of rigorous assay development with graphene‐enhanced SPR and portable translation into gFET devices positions this strategy as a strong foundation for future point‐of‐care diagnostics.

## 2 Results

### 2.1 Theoretical Concept and Assembly of Optical and Electrical Biosensors

**Figure 1:**
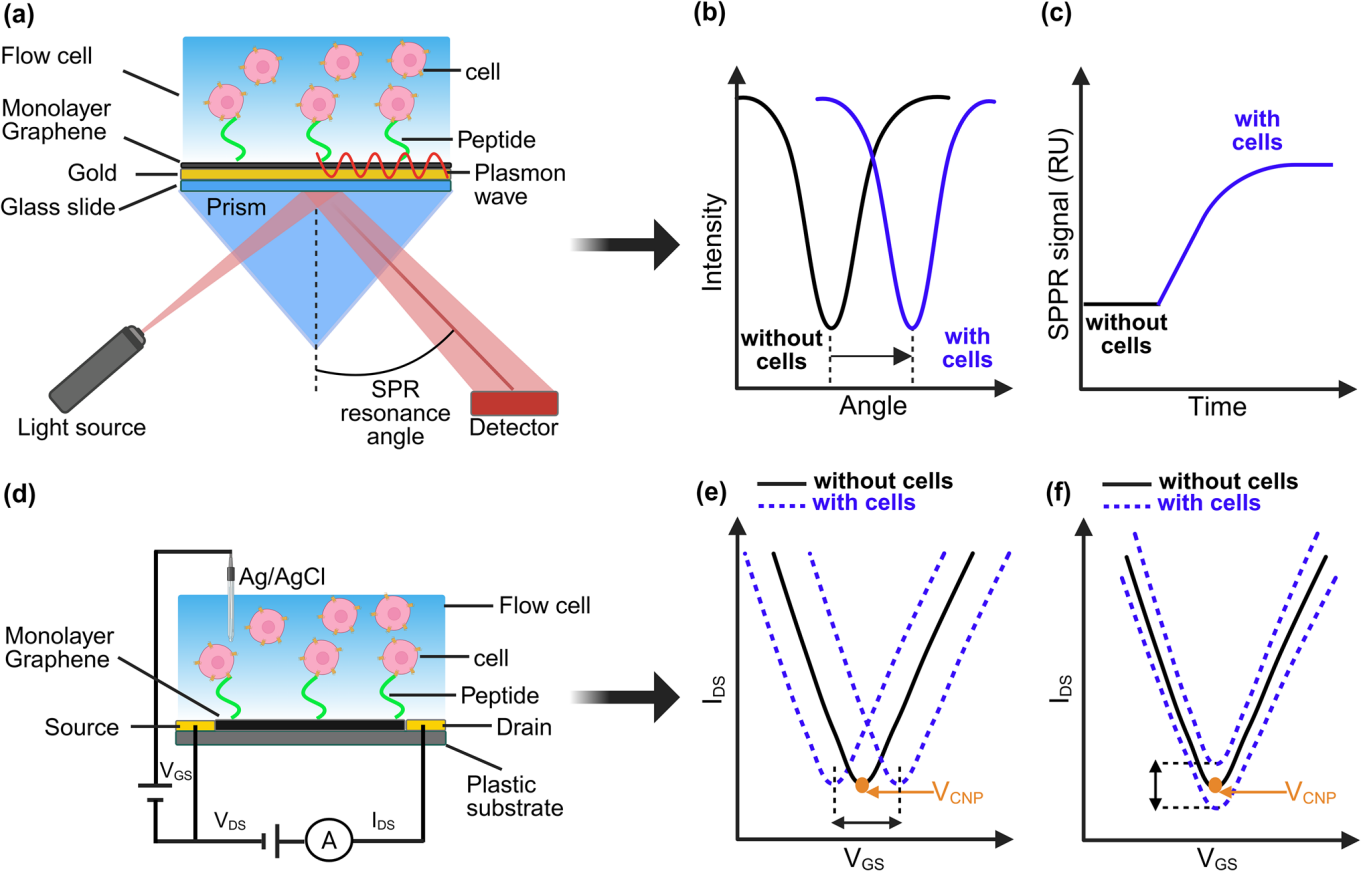
Graphene‐based biosensing platforms for detecting receptor‐peptide interactions: a) Schematic of graphene‐enhanced surface plasmon resonance (SPR) using a graphene‐coated gold substrate functionalized with peptides. Binding of receptor‐expressing cells alters the plasmon resonance angle. b) Representative angular SPR spectra showing a redshift upon cell binding (blue curve). c) SPR sensorgram illustrating the real‐time signal increase during cell association. d) Graphene field‐effect transistor (gFET) device with a peptide‐functionalized graphene channel for electrical detection of binding events. e) Transfer characteristics of gFETs, where cell binding induces a shift in the charge neutrality point (V_CNP_) and changes in drain current (I_DS_), reflecting charge transfer and surface potential modulation.

In our study, we implemented two complementary biosensing platforms. The optical biosensor was based on the Kretschmann configuration^[^ ^34^ ^]^ of SPR, incorporating a monolayer graphene coating on a gold sensor chip to enhance sensitivity and stability (**Figure** **1**a–c).^[^ ^7^, ^35^ ^]^ The electrical biosensor adopted a liquid‐gated gFET configuration, using a Ag/AgCl reference electrode (Figure 1d–f).^[^ ^15^ ^]^ More details on the measurement setup and surface characterization are provided in Figure S1 (Supporting Information).

**Figure 2:**
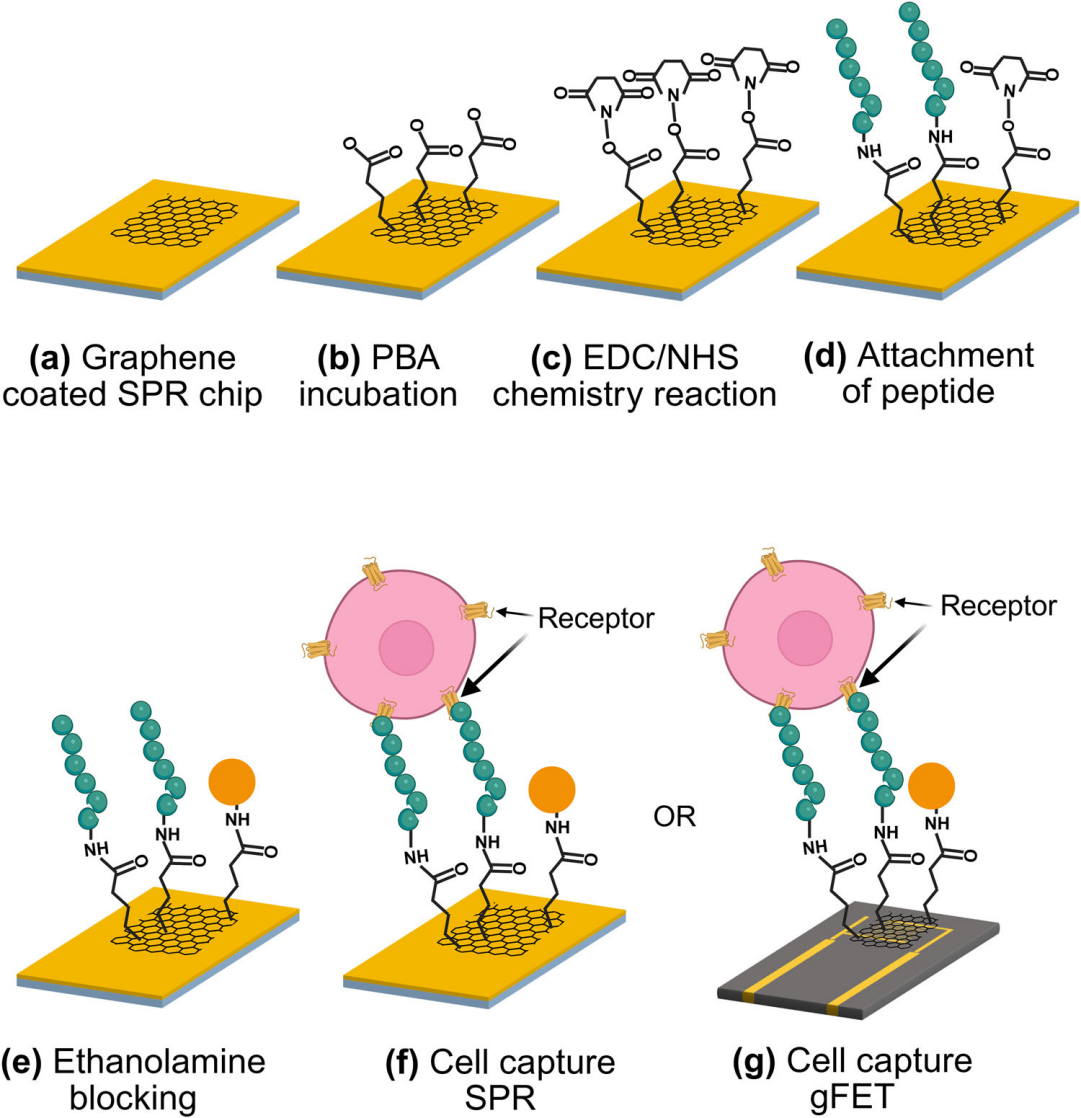
Stepwise functionalization of graphene biosensing assays: a) Transfer of CVD‐grown monolayer graphene onto sensor substrates. b) Immobilization of 1‐pyrenebutyric acid (PBA) linker via π–π stacking. c,d) Activation of carboxyl groups by EDC/NHS chemistry, covalent attachment of amine‐terminated peptides, and ethanolamine blocking of unreacted sites. f) Receptor‐mediated cell capture on graphene‐enhanced SPR chips. g) Receptor‐mediated cell capture on liquid‐gated graphene field‐effect transistor (gFET) sensors.

Both platforms employed an identical assay protocol (**Figure** **2**). Peptides were immobilized onto the graphene surfaces via amine coupling chemistry,^[^ ^36^ ^]^ enabling direct comparison across modalities. Biosensor performance was evaluated using different cellular models. Specific peptide‐cell interactions produced a measurable increase in the SPR signal (Figure 1b,c), whereas in the electrical biosensor, binding events resulted in a shift of the charge neutrality point (V_CNP_) or a change in channel conductivity (I_DS_) (Figure 1e,f).

The schematic in Figure 2 illustrates the stepwise functionalization workflow used for both optical and electrical biosensing assays targeting receptor‐mediated cell capture. First, CVD‐grown monolayer graphene was transferred onto sensor chips using a wet transfer method.^[^ ^37^, ^38^ ^]^

The graphene surface was noncovalently modified with 1‐pyrenebutyric acid (PBA) via π‐π stacking to introduce carboxyl groups,^[^ ^39^ ^]^ which were subsequently activated by EDC/NHS to generate reactive esters.^[^ ^40^ ^]^ Amine‐terminated peptides were then coupled through amide bond formation, followed by ethanolamine quench to block residual esters. For WKYMVm‐NH_2_, coupling can occur at either the α‐amine or Lys ε‐amine, and for f‐MEQQNK, N‐formylation of the N‐terminus inherently directs coupling to Lys ε‐amine. Stepwise schematics of peptide functionalization is shown in Figure 2a–e.

Because the study employs a dual‐platform approach, identical peptide functionalization procedures were applied to both SPR and gFET sensors. Sensor responses following each functionalization step are shown in Figure S2 (gFET) and Figure S3 (SPR). Detailed protocols, including peptide selection and step‐by‐step immobilization procedures for both platforms, are provided in Section S3 (Supporting Information). Successful surface modification was further confirmed by contact‐angle measurements (Figure S6, Supporting Information).

### 2.2 Detection of Peptide Interaction with FPR2‐Expressing HEK293T Cells

To establish a receptor‐targeted biosensor, we first needed to identify suitable peptides for target and control. For this purpose, we used transiently transfected HEK293T cells that overexpressed individual members of the FPR family (FPR1‐3). As the target peptide, we selected the W‐peptide (WKYMVm‐NH_2_) amongst many FPR2 activators, because W‐peptide is a well‐characterized, sensitive FPR activator known from literature that additionally provides off‐the‐shelf availability. The selective binding was also confirmed by optical imaging (Figure S11, Supporting Information). Consistent with prior reports,^[^ ^4^, ^28^, ^29^ ^]^ this peptide induced intracellular calcium flux in cells expressing FPR2 (Figure S7a, Supporting Information) and FPR1 (Figure S7c, Supporting Information) at low nanomolar concentrations. Together, these attributes make W‐peptide in our opinion a suitable choice for the detection of FPR2 and the method's reproducibility. As a negative control we used the peptide f‐MEQQNK, a natural peptide derived from bacteria, that despite carrying the characteristic structural properties of FPR2 activators^[^ ^4^, ^41^ ^]^ such as a formyl‐modification and an N‐terminal methionine residue, does not activate FPR2 or other FPR subtypes (Figure S7b,d, Supporting Information). Moreover, due to its Lysine residue at the most C‐terminal position, f‐MEQQNK enables for easier functionalization and surface coupling which made it stood out from other non‐activators. Based on these results, we selected WKYMVm‐NH_2_ as the target peptide (Target‐Pep) and f‐MEQQNK as the control peptide (Control‐Pep). The peptide structures are shown in Figure S4 (Supporting Information).

**Figure 3:**
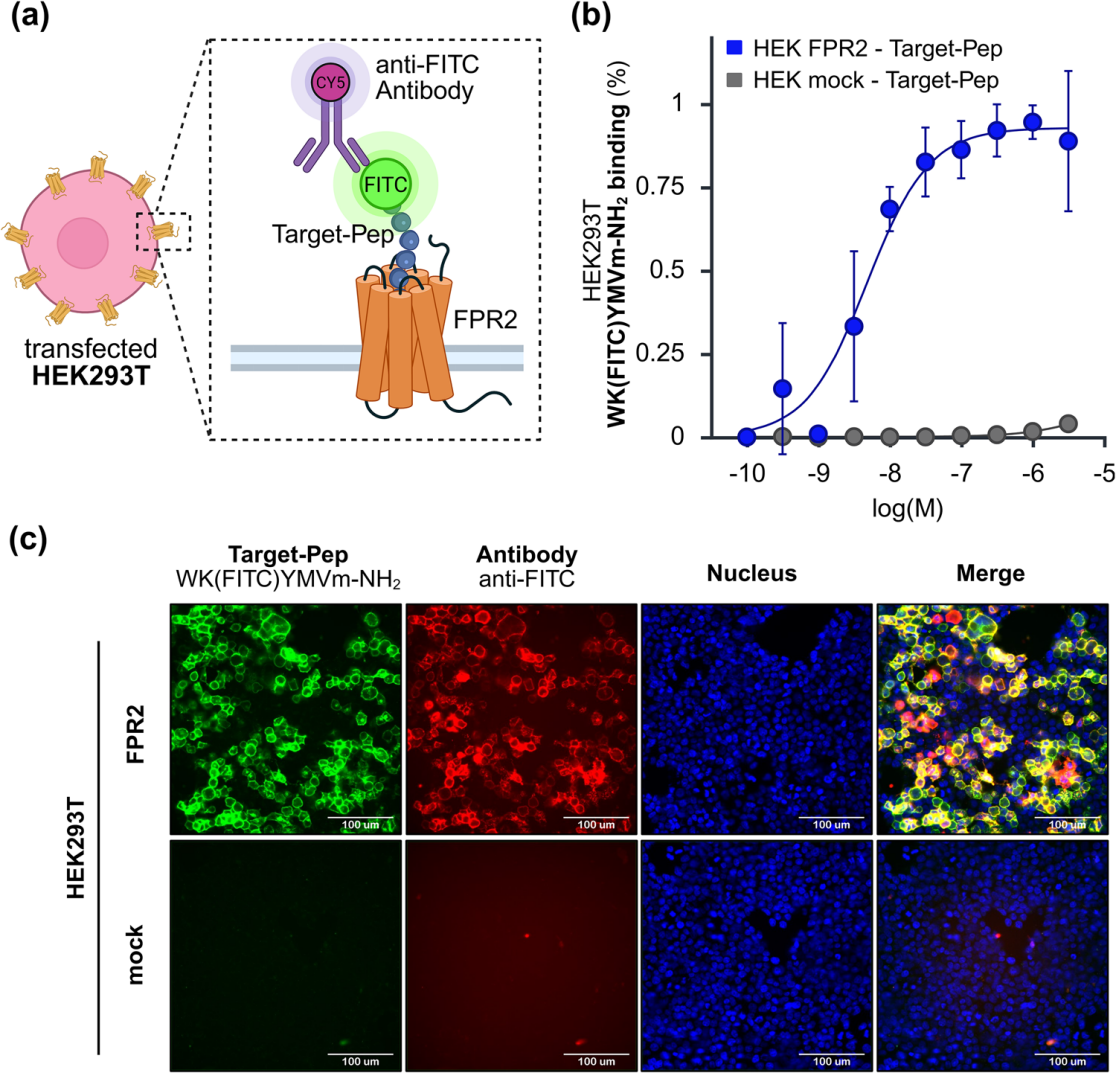
Evaluation of FPR2 as a biosensor target: a) Schematic of the assay principle. The FITC‐labeled target peptide (WK(FITC)YMVm‐NH_2_) binds to FPR2, and accessibility of the FITC group is assessed using an anti‐FITC antibody. b) Binding kinetics of the FITC‐labeled target peptide on FPR2‐transfected HEK293T cells (HEK FPR2) (n = 3) compared with mock‐transfected controls (HEK mock) (*n* = 3). Binding is shown as the percentage of ligand‐positive cells and normalized to the maximum value of each dataset. Data represent mean ± SD. c) Representative confocal images of HEK cells incubated with FITC‐labeled target peptide (1 µm) followed by staining with anti‐FITC antibody (5 µg mL^−1^). Nuclei were counterstained with Hoechst 33 342 (20 µm). Scale bar: 100 µm.

We next examined whether the target peptide would retain its receptor specificity when lysine amino residue in WKYMVm‐NH_2_ was modified (**Figure** **3**a), as required for subsequent biosensor coupling (Section S3, Supporting Information). To test this, we introduced a fluorescein isothiocyanate (FITC) group to the lysine because this allowed us to evaluate general effects of a chemical modification at this site on the interaction with the receptors. Next, the FITC group allowed us to directly monitor the binding of the peptide to the different FPR receptors. The binding assays confirmed that FITC‐functionalization did not impair activity, as FPR2 (Figure 3b) and FPR1 (Figure S8a, Supporting Information) remained responsive.

Interestingly, when an anti‐FITC antibody was applied after peptide binding, a differential accessibility pattern was observed: the antibody stained FITC‐labeled peptides bound to FPR2 (Figure 3c) but not those bound to FPR1 (Figure S8b, Supporting Information). This suggests that the lysine residue of the target peptide remains exposed when bound to FPR2 but is sterically constrained within the binding pocket of FPR1.

Together, these results highlight FPR2 as a particularly suitable receptor for biosensor development, as WKYMVm‐NH_2_ retains high activity and provides an accessible functionalization site for coupling without loss of specificity.

### 2.3 Specific Detection of FPR2‐Peptide Interactions Using Graphene‐Enhanced SPR

**Figure 4:**
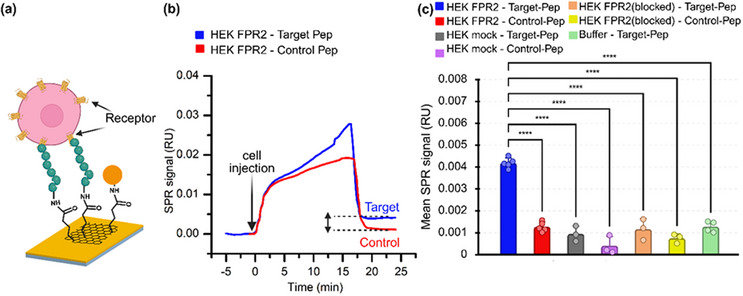
SPR analysis of FPR2‐peptide interactions in HEK293T cells: a) Schematic illustrating the binding of FPR2‐expressing HEK293T cells to surface‐immobilized peptides. b) Representative SPR sensorgram showing real‐time binding of FPR2‐expressing cells to the target peptide (blue) versus the Control peptide (red). c) Specificity of mean SPR responses to the target peptide compared with six different negative controls. Data are shown as mean ± SD (*n* = 3–5 independent experiments: HEK FPR2‐Target‐Pep and HEK FPR2‐Control‐Pep, *n* = 5; Buffer‐Target‐Pep, *n* = 4; all other groups: *n* = 3). Statistical significance between groups was assessed using a one‐way ANOVA. All significant pairwise differences were **** (*p* < 0.0001).

Building on our previous findings^[^ ^12^ ^]^ and in agreement with published reports,^[^ ^7^, ^35^ ^]^ integration of graphene onto the SPR interface enhances performance by increasing sensitivity and stabilizing the baseline. Accordingly, we employed graphene‐enhanced SPR to evaluate interactions between FPR2‐expressing HEK293T cells and immobilized peptides (**Figure** **4**a). The BioNavis SPR system enables two experimental channels in parallel,^[^ ^42^ ^]^ allowing simultaneous testing of the target peptide (WKYMVm‐NH_2_) and the Control peptide (f‐MEQQNK) (Figure S5a, Supporting Information). Both peptides were immobilized via amine coupling, in which the lysine residue reacts with the surface NHS ester.

Cells were injected into both channels at a speed of 10 µL min^−1^. The injected cell suspension contained only ≈100 cells mL^−1^; thus, over the 20‐min injection, ≈20 cells interacted with each channel. Despite this low cell number, strong binding was observed on the target peptide channel, which produced a steep association slope (Figure 4b, blue curve). In contrast, Control peptide binding remained minimal. During the dissociation phase, buffer injection removed weak, nonspecific interactions, leading to a stable baseline signal (Figure 4c). Quantitatively, FPR2‐expressing cells yielded an SPR signal on target peptide surfaces that was approximately fourfold higher than on Control peptide surfaces. These results confirm the specificity and sensitivity of the peptide‐receptor interaction, even under conditions of very low cell numbers.

### 2.4 Control Validation and Optical Confirmation

Multiple stringent controls were performed to confirm the specificity of the observed SPR responses. HEK293T cells without FPR2 expression, expectedly, produced no strong interaction with the surfaces that were coated with any of both peptides (Figure 4c, gray and purple bars). In another experiment, we blocked the specific binding site of the FPR2 receptor in HEK293T cells that were expressing this receptor by pre‐incubatiing them with the target peptide (3 µm) prior to injection into the SPR. This treatment again resulted in a very low signal comparable to previous controls (Figure 4c, orange and yellow bar).

Background responses from the running buffer (C1) were also minimal (Figure 4c, green bar) and likely reflect bulk refractive index contributions from glucose (1.8 g L^−1^) in the buffer. Collectively, these controls reduced the strong FPR2‐dependent responses to baseline levels, demonstrating that our signal arises from the specific interaction of receptors on the surface of living cells with peptides bound to the graphene surface.

As an orthogonal validation of the adherence of FPR2 expressing cells on peptide coated surfaces, fluorescence microscopy was performed on graphene‐coated slides functionalized with either target peptide or control peptide and seeded with GFP‐transfected HEK293T cells. A third condition included mock‐transfected cells seeded on target peptide surfaces (Figure S11, Supporting Information).

Mosaic images revealed clear enrichment of FPR2‐expressing cells on slides functionalized with target peptide in comparison to slide with control peptide or mock‐transfected cells. Quantitative cell counting confirmed significantly higher adherence of cells in the target peptide condition (Figure S11a, Supporting Information). Together, these results demonstrate that even at very low cell numbers (≈20 cells), FPR2‐specific interactions can be robustly detected.

### 2.5 Translation to Graphene Field‐Effect Transistor (gFET) Biosensors

**Figure 5:**
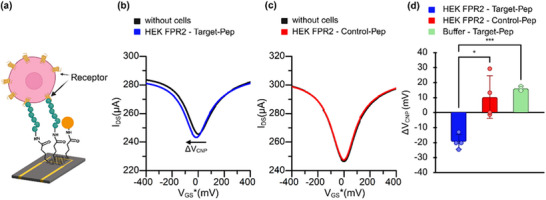
gFET analysis of FPR2‐peptide interactions in HEK293T cells: a) Schematic illustrating FPR2‐expressing HEK293T cells interacting with peptide‐functionalized graphene FET sensors. b) Transfer curves showing an n‐type (leftward) shift in charge neutrality point (ΔV_CNP_, indicated by the arrow) upon binding of FPR2‐expressing cells (HEK‐FPR2) to the target peptide (blue curve), indicating receptor‐specific interaction. c) Minimal V_CNP_ shift observed when FPR2‐expressing cells interact with the control peptide (red curve), indicating low nonspecific binding. b,c) Transfer curves are plotted versus the gate voltage referenced to the baseline CNP for clarity (V_GS_* = V_GS_‐V_CNP, baseline_) d) Quantification of V_CNP_ shifts across experimental replicates. ΔV_CNP_ is defined as ΔV_CNP_ = V_CNP, with cells_ − V_CNP, baseline_; negative values correspond to leftward (n‐type) shifts, while positive values correspond to rightward (p‐type) shifts. Bars represent mean ± SD. Statistical significance was assessed using Welch´s ANOVA with Dunnett's T3 post‐hoc tests (adjusted p‐values), *n* = 4 for cell‐based measurements and n = 3 for the buffer control. Welch's ANOVA: W(2, 4.46) = 84.24, p = 0.000287. Dunnett's T3 (adjusted): Target versus Control, p = 0.0413; Target versus Buffer, p = 0.000280. Significance levels: ns, p ≥ 0.05; ^*^, p < 0.05; ^**^, p < 0.01; ^***^, p < 0.001.

Having validated the assay using SPR, we next applied the same workflow to a gFET platform (**Figure** **5**a). Two gFET devices, each equipped with independent in‐house fluidic modules, were operated under the same molecular and buffer flow conditions used in the SPR experiments. Target and Control peptides were covalently immobilized on the graphene channels via amine‐coupling chemistry (Figure S5b, Supporting Information). Baseline transfer characteristics (V_GS_‐I_DS_) were recorded in buffer after immobilization and ethanolamine blocking (black curves, Figure 5b,c).

Introduction of FPR2‐expressing HEK293T cells (10 µL min^−1^), followed by buffer flushing, produced a clear electrical response. When plotted versus the referenced gate voltage (V_GS_* = V_GS_‐V_CNP, baseline_), devices functionalized with the target peptide showed a pronounced negative shift of charge neutrality point (V_CNP_) relative to baseline, along with reduced channel conductivity (Figure 5b, blue curve). In contrast, Control peptide devices displayed only a negligible positive shift in V_CNP_ shift (Figure 5c, red curve). These opposite responses provide clear electrical discrimination between specific and nonspecific interactions.

Across four experiments, cells interacting with the target peptide produced ΔV_CNP_ shifts of −13.1, −20.4, −24.8, and −20.4 mV, with a mean ΔV_CNP_ of −19.65 mV (Figure 5d, blue bars). Control peptide devices showed shifts of −1.5, 13.1, 29.1, and 0 mV (Figure 5d, red bars), with a mean ΔV_CNP_ of 10.18 mV, consistent with nonspecific physical adsorption or buffer‐related effects. Similarly, the buffer‐only control exhibited a mean ΔV_CNP_ shift of 16 mV toward higher gate voltage (Figure 5d, green bar).

As an additional parameter, the drain current at the charge neutrality point was analyzed and plotted in Figure S10a (Supporting Information), confirming significant differences between the target and control.

To evaluate sensor performance, we applied a fixed threshold of −7.2 mV, chosen as the midpoint between the least‐negative target and the lowest Control value (Section S8, Supporting Information). Using this threshold, the confusion matrix was TP = 4, FP = 0, FN = 0, TN = 7, yielding Precision = 1.00, Recall/Sensitivity = 1.00, and F1‐score = 1.00. Exact binomial 95% confidence intervals were Sensitivity 100% (39.8–100%) and Specificity 100% (59.0–100%). These decision‐level results align with the group differences observed in Welch's ANOVA (Figure 5d) and are consistent with the expected leftward ΔV_CNP_ shifts for target peptide binding.

### 2.6 The Biosensor Approach enables Detection of FPR2 on Neutrophils from Human Blood Samples

Human neutrophils are the most abundant innate immune cells in human blood and express the highest levels of FPR1 and FPR2 receptors on their surface.^[^ ^24^, ^26^, ^27^, ^43^, ^44^ ^]^ In immunity, they play a central role in host defence by recognizing N‐formylated peptides derived from bacteria or from mitochondria of damaged host cells.^[^ ^25^, ^26^, ^27^, ^31^, ^41^ ^]^ In our earlier experiments, we used FPR2‐transfected HEK293T cells as a model to study peptide‐receptor interactions of the biosensor, providing proof that detection of a G‐protein coupled receptor on the surface of living cells via SPR or gFET is feasible. We next sought to determine whether these principles could be extended to primary human blood cells, such as neutrophils, which natively express FPR2.

**Figure 6:**
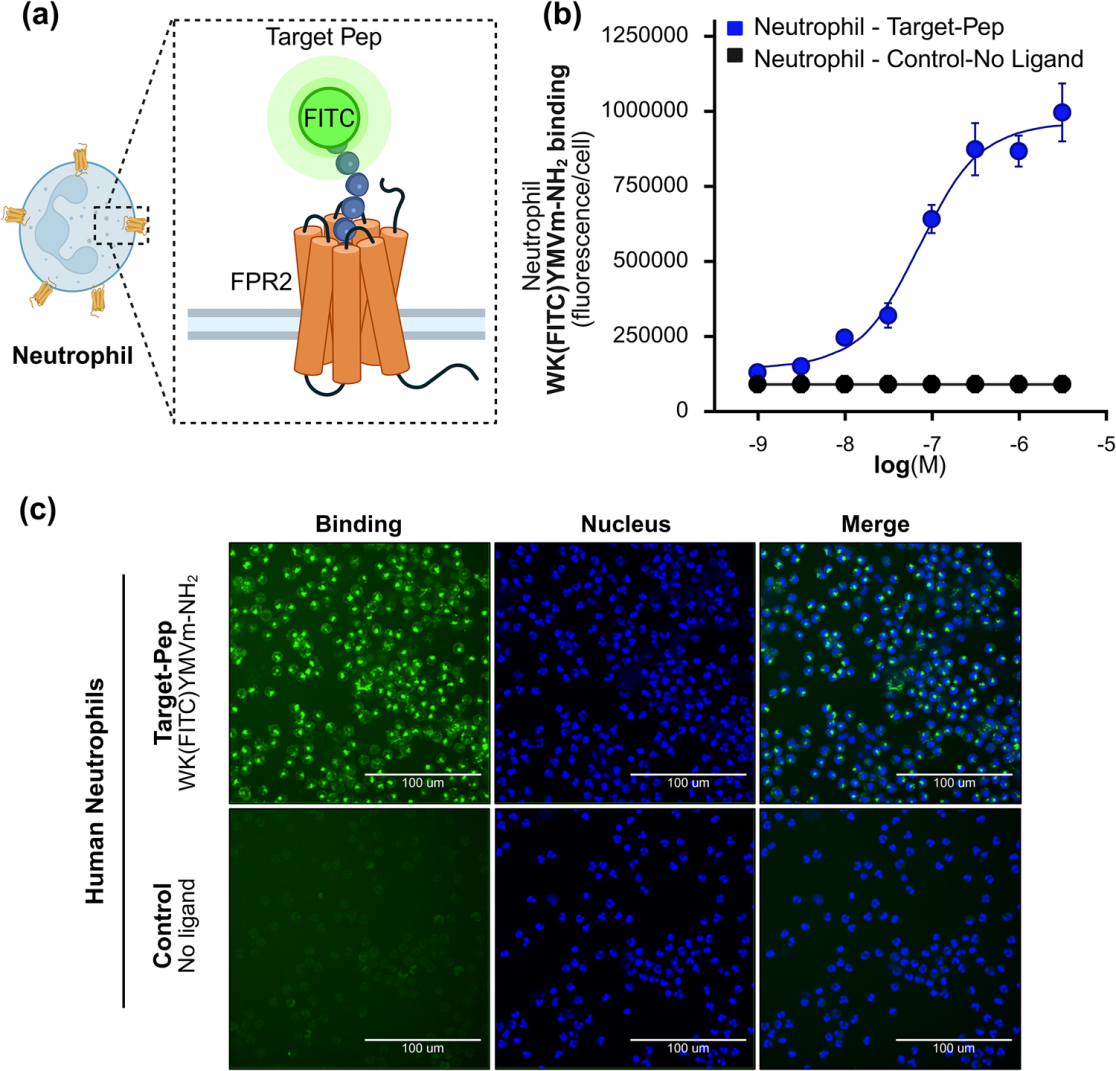
Target peptide enables specific binding to primary human neutrophils: a) Schematic illustrating the binding of the fluorescently labeled target peptide WK(FITC)YMVm‐NH_2_ to FPR2 on human neutrophils. b) Binding kinetics of WK(FITC)YMVm‐NH_2_ to freshly isolated neutrophils (*n* = 2). Binding is expressed as fluorescence intensity per cell. Background control was measured in the absence of ligand. Data are shown as mean ± SD. c) Representative confocal images of neutrophils incubated with WK(FITC)YMVm‐NH_2_ (1 µm). Nuclei were stained with Hoechst 33 342 (20 µm). Scale bar: 100 µm.

To this end, we examined the interaction of the target peptide with freshly isolated human neutrophils (**Figure** **6**a). As expected, the target peptide activated neutrophils (Figure S9, Supporting Information), and its FITC‐labeled analogue bound efficiently to these innate immune cells (Figure 6a–c). These results confirmed that the biosensor's target peptide (W‐Peptide) can also capture primary neutrophils.

**Figure 7:**
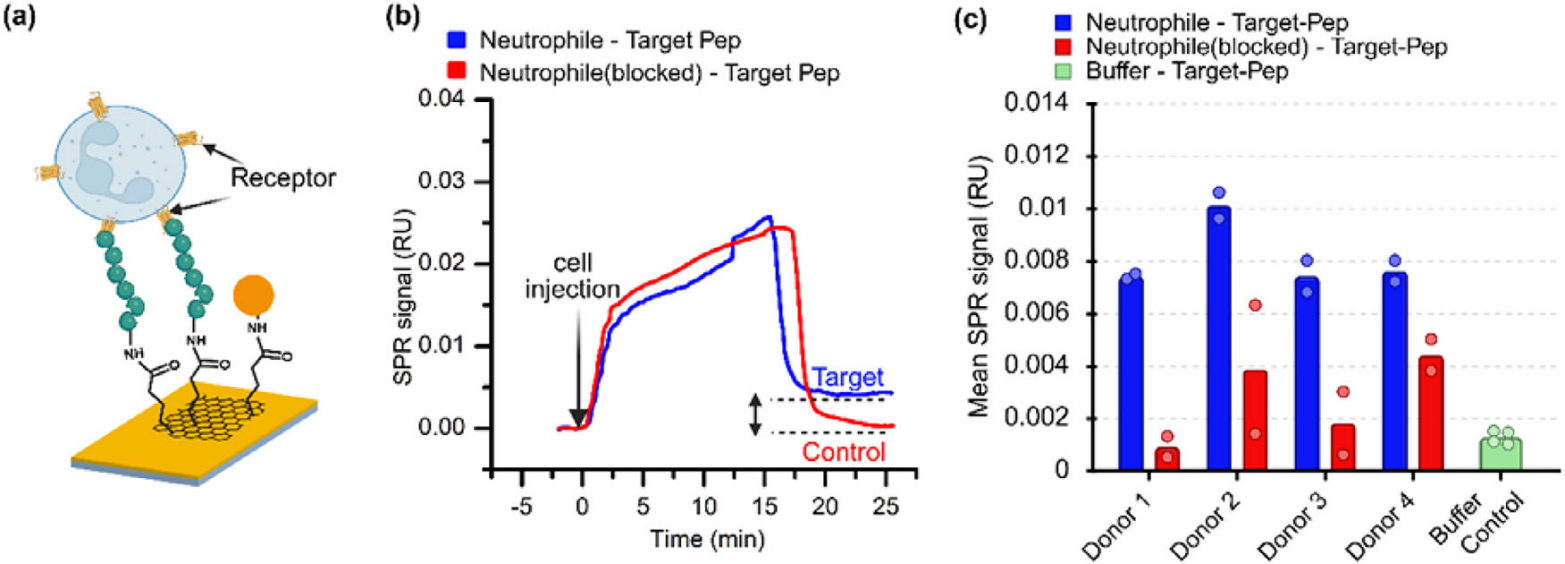
SPR analysis of FPR2‐mediated peptide binding in primary human neutrophils: a) Schematic illustration of neutrophils interacting with immobilized target peptide via surface FPR2 receptors. b) Representative SPR sensorgram showing strong binding of neutrophils to the target peptide (blue) and reduced binding when receptors were pre‐blocked with target‐peptide (red). c) Quantification of SPR signals from neutrophils isolated from two donors, demonstrating decreased binding upon receptor blocking and confirming receptor‐specific interactions.

In comparison to the overexpressing HEK293T model cells, neutrophils have much more heterogeneous surface and lower FPR2 abundance. for SPR analysis a concentration of 10 000 neutrophils/mL at a flow rate of 10 µL min^−1^ was found optimal. **Figure** **7**a shows a schematic of immune cells interacting with immobilized peptide, while Figure 7b presents a representative sensogram. Neutrophils exhibited strong binding to target peptide (blue curve), in contrast to the minimal binding observed when the specific binding site of FPR2 was blocked by preincubation with the target peptide (3 µm) prior to the experiment (red curve). This receptor‐blocking control was selected as the most stringent condition to demonstrate that the observed binding is specifically mediated by the target peptide‐FPR2 interaction. The initial sharp increase in SPR signal (Figure 7b) reflects neutrophils entering the flow chamber and contacting the sensor surface. The subsequent steady rise corresponds to sustained cell‐peptide interactions, whereas the rapid decline reflects the dissociation of unbound cells.

In the control channel, the SPR signal returned to baseline, whereas in the target channel it remained elevated, indicating that cell binding occurred specifically through target peptide‐ FPR2 interactions (Figure 7b). To confirm reproducibility, neutrophils from four independent donors were analyzed, with duplicate measurements performed for each donor (Figure 7c). Untreated neutrophils consistently produced significantly higher binding signals compared to receptor‐blocked neutrophils, confirming receptor‐specific interactions (Figure 7c).

**Figure 8:**
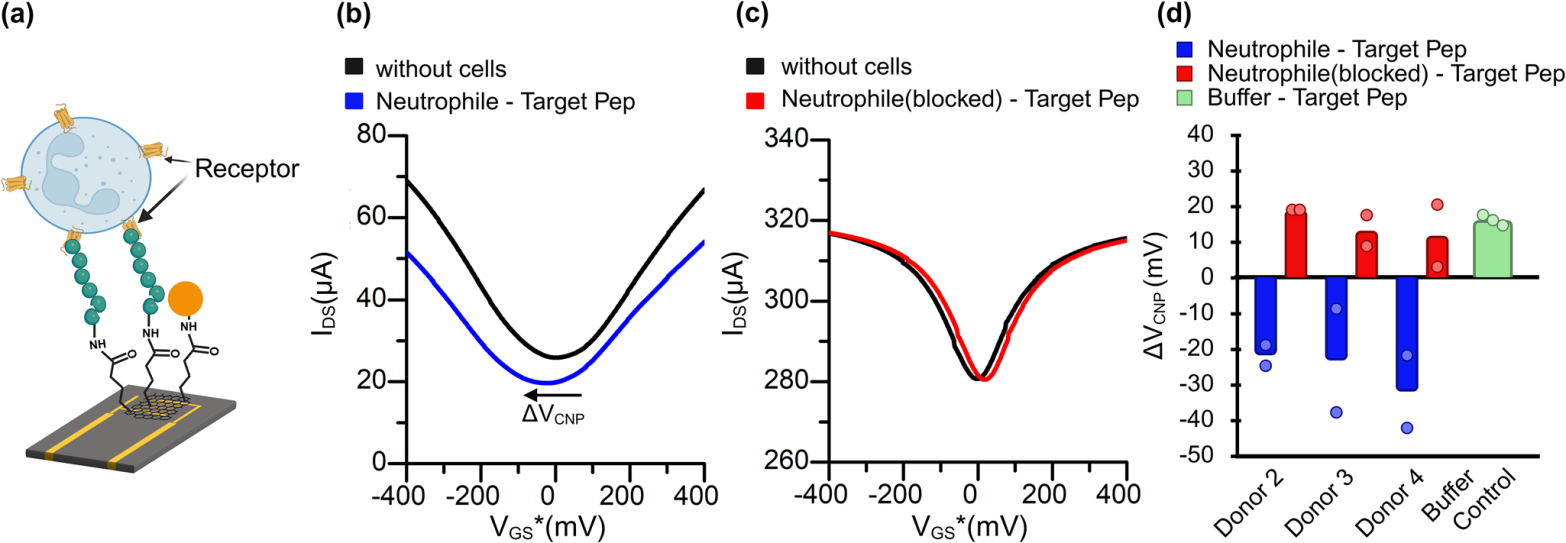
gFET analysis of FPR2‐mediated peptide binding in primary human neutrophils: a) Schematic illustrating neutrophils binding to immobilized target peptide via surface FPR2 receptors. b) Transfer curves showing a leftward (n‐type) shift in V_CNP_ upon neutrophil binding to the target peptide, as indicated by the arrow. c) Neutrophils with blocked FPR2 showed minimal V_CNP_ shift, confirming receptor‐specific binding. b,c) Transfer curves are plotted versus gate voltage referenced to baseline CNP (V_GS_* = V_GS_‐V_CNP, baseline_) d) Summary of V_CNP_ shifts from neutrophils isolated from two donors, showing consistent receptor‐specific signal only in unblocked cells.

Similar biosensing experiments were conducted using an electrical biosensor (gFET) as shown in **Figure** **8**. Two gFET sensor chips integrated with a fluidic system were employed to mimic molecular or buffer flow, analogous to the SPR setup. Baseline I_DS_‐V_GS_ curves were first recorded in buffer after immobilizing either the target or control peptide (black curves, Figure 8b,c). At this stage, no neutrophils were present in the chambers.

For analysis, the gate‐source voltage was referenced to the baseline charge neutrality point, defining (V_GS_* = V_GS_‐V_CNP, baseline_); all curves in Figure 8b,c are plotted versus V_GS_*. Neutrophils (10 000 cells mL^−1^) were introduced into both devices at a flow rate of 10 µL min^−1^. After cell injection, buffer was flushed through the chambers to remove unbound cells, and a second I_DS_‐V_GS_* measurement was performed. In Figure 8b, the blue sweep shows a pronounced leftward (n‐type) shift in V_CNP_ relative to baseline, accompanied by a marked decrease in conductivity, indicating successful peptide‐cell binding. By contrast, the red sweep in Figure 8c shows a rightward V_CNP_ shift with no conductivity decrease, consistent with nonspecific interaction or lack of binding under control conditions.

Consistent with the SPR results, replicates from three donors (Donors 2–4, corresponding to Figure 7) showed significant leftward V_CNP_ shifts (ΔV_CNP_ < 0) for target‐functionalized sensors, whereas control sensors exhibited rightward shifts (ΔV_CNP_ > 0) (Figure 8d). These results confirm receptor‐specific FPR2‐peptide interactions and demonstrate that the gFET can detect primary human neutrophils at native receptor expression levels. Notably, experiments for all the three donors were conducted individually on the same day using both SPR and gFET platforms.

Using a fixed ΔV_CNP_ threshold of −2.9 mV, gFET responses were analyzed for three donors with two technical replicates per donor. True positives (TP), false positives (FP), false negatives (FN), and true negatives (TN) were: TP = 6, FP = 0, FN = 0, TN = 9, yielding a Precision of 1.00, Recall/Sensitivity of 1.00, and F1‐score of 1.00. Exact binomial 95% confidence intervals were 100% (54.1–100%) for Sensitivity and 100% (66.4–100%) for Specificity.

All three donors produced target signals exceeding the threshold when replicates were aggregated (median ΔV_CNP_ per donor), consistent with the expected leftward ΔV_CNP_ shift for FPR2 engagement. Because replicates are clustered within donors and the sample size is small, between‐group ANOVA was not performed; the paired analysis approach described in the Methods section is more appropriate for this experimental design.

These results confirm the sensor's robust ability to discriminate between specific target‐induced signals and nonspecific responses. In addition to evaluating V_CNP_ shifts, we also examined the drain‐source current (I_DS_) at the charge neutrality point (V_CNP_). Upon binding to target peptide (WKYMVm‐NH_2_), primary neutrophils generated a clear negative change in drain‐source current (Figure S7b, Supporting Information), whereas receptor blocking suppressed or even reversed the signal. This supports the presence of a receptor‐dependent electrical signature, consistent with the receptor‐mediated Ca^2^⁺ flux responses observed in Figures S7 and S8 (Supporting Information).

## 3 Discussion

We developed a proof‐of‐principle peptide‐functionalized biosensor based on monolayer graphene and evaluated it using two complementary platforms: liquid‐gated gFETs and SPR. Applying identical surface chemistry across both platforms allowed direct comparison of transduction without altering biological conditions. FPR2‐transfected HEK293T cells provided a controlled receptor expression system, while primary human neutrophils represented physiologically relevant immune cells. Across both platforms, the resulting response patterns consistently indicate a highly specific and sensitive interaction between FPR2 on the cell surface and the target peptide (WKYMVm‐NH_2_) on the biosensor.

SPR, a well‐established optical technique, was used for assay development and validation. FPR2‐expressing HEK293T cells exhibited pronounced binding to the target peptide, whereas mock‐transfected cells, control peptide surfaces, and receptor‐blocked cells remained at baseline. Fluorescence microscopy confirmed enhanced cell adhesion on target peptide surfaces. Neutrophils from four donors displayed reproducible, sensitive binding that was abolished upon receptor blockade. While SPR provides rigorous validation, its reliance on complex instrumentation limits portability and scalability.

In contrast, gFET measurements were designed with point‐of‐care applications in mind, offering portability, scalability, and rapid readouts suitable for clinical or field settings. Receptor‐ligand engagement induced n‐type shifts in the charge neutrality point for both FPR2‐transfected HEK293T cells and untreated neutrophils. Pre‐blocked receptors and buffer‐only controls produced negligible or weakly opposite responses. These electrical changes are consistent with interfacial electrostatics at the cell‐graphene interface: peptide engagement promotes receptor clustering and activation, including β‐arrestin recruitment,^[^ ^30^ ^]^ which brings charged or dipolar groups close to the graphene lattice, modulating its carrier density.^[^ ^16^ ^]^ Acidic residues in the FPR2 pocket, together with protonated amines on the ligand, likely generate local dipoles that increase electron density,^[^ ^45^, ^46^ ^]^ yielding an effective n‐type gating.

Under blocked conditions, gFETs showed an apparent p‐type shift or negligible n‐type response (Figures 5d and 8d), confirming that the pronounced n‐type signal is specifically mediated by FPR2‐ligand interactions. FPR2‐positive cells exposed to target peptide also showed a decrease in drain current (I_DS_) at the charge neutrality point (Figure S10a,b, Supporting Information), consistent with the leftward shift of the (V_CNP_) and the transition toward an insulating cell‐graphene contact. Residual variability likely reflects differences in peptide coverage, receptor density, or transfection efficiency. Applying a simple threshold to V_CNP_ shifts cleanly separated target from controls, yielding Precision, Recall, and F1‐score of 1.0 in both HEK293T and neutrophil assays.

In this biosensing architecture, monolayer graphene transduces the signal, while peptide ligands confer molecular selectivity for cell capture. Cell binding couples the membrane to the graphene lattice, modulating carrier density via the membrane's net surface charge, interfacial dipole orientation, and local ionic distribution.^[^ ^11^ ^]^ High binding affinity of WKYMVm‐NH_2_ within the FPR2 pocket, supported by hydrophobic and hydrogen‐bond interactions, promotes receptor clustering and conformational rearrangements, further modulating interfacial electrostatics.^[^ ^47^ ^]^ These perturbations produce reproducible conductance changes and shifts in the charge neutrality point, enabling label‐free electrical detection of cell‐surface interactions. Beyond methodological validation, these findings may also have biomedical relevance. Neutrophils and their receptors, including FPR2, play key roles in inflammation and are implicated in immune dysregulation and sepsis.^[^ ^27^, ^48^, ^49^ ^]^ Label‐free, quantitative detection of immune cells and receptors using peptide‐functionalized graphene could enable POC immune profiling.

Our sensing principle combining functionalized peptides with graphene aligns with conventional detection methods such as flow cytometry and ELISAs. ELISAs provide sensitive, robust quantification but typically analyze bulk lysates relative to total protein. Flow cytometry offers similar sensitivity and adds single‐cell resolution but requires expensive instrumentation. Both methods rely on antibodies, which can be costly, limited in availability, prone to nonspecific binding, and dependent on specific epitopes. Peptides circumvent many of these issues: they are inexpensive, stable, and can achieve high‐affinity, rapid and firm binding, as exemplified for FPR2‐detection.^[^ ^50^ ^]^

gFETs provide a miniaturizable, electrical readout suitable for real‐time, label‐free, point‐of‐care detection with minimal sample handling. While SPR provides rigorous validation, gFETs represent the preferred modality for translational deployment due to their practical advantages in low‐resource or decentralized settings.

The graphene‐peptide sensor shows strong potential for point‐of‐care applications. Its room‐temperature operation, stable non‐enzymatic chemistry, and electrical readout suggest compatibility with small, battery‐ or self‐powered devices. Target‐Control pairing could be implemented on a single disposable cartridge with internal referencing, and pre‐functionalized, dry‐stored cartridges with capillary‐driven flow could eliminate the need for pumps and reduce assay complexity for the end user. Immediate applications may include rule‐in/rule‐out assays for disease‐relevant receptors or pathogen proteins, while near‐term translation would require analytical and clinical validation, as well as scalable manufacturing of graphene cartridges.

Overall, our platform demonstrates sensitivity, robustness, and reproducibility at low cell numbers. Its modular peptide functionalization and versatile graphene transduction indicate that this approach could be generalized across receptor‐ligand systems, supporting future studies in diagnostics, immune profiling, and early sepsis monitoring.

## 4 Experimental Section

### Materials

Monolayer graphene was purchased from Graphenea. 1‐Pyrenebutyric acid, N‐hydroxysuccinimide (NHS), 1‐ethyl‐3‐(3‐dimethylaminopropyl) carbodiimide (EDC), and ethanolamine were obtained from Sigma–Aldrich. All chemicals were of analytical grade or higher. C1 assay buffer (130 mm NaCl, 10 mm HEPES, 5 mm KCl, 2 mm CaCl_2_, 5 mm glucose, pH 7.4) was purchased from Carl Roth.

### Multiparametric SPR (MP‐SPR)

SPR experiments were performed using an MP‐SPR Navi 200 (BioNavis Ltd., Tampere, Finland) equipped with gold‐coated chips (2 nm Cr / 50 nm Au; BioNavis Ltd., Finland) and a flow cell with a 1 µL channel volume. For this study, the gold chips were modified with monolayer graphene. Measurements were conducted in angular scan mode at 670 nm to monitor real‐time shifts in the SPR minimum. Before each run, the injection system and flow channels were thoroughly washed and filled with running buffer. The sensor temperature was maintained at 22 °C, and the flow rate was adjusted according to the binding experiment step.

### Graphene‐Enhanced SPR Chip Preparation

Monolayer graphene sheets were transferred onto gold SPR chips using a standard wet‐transfer method.^[^ ^15^, ^37^, ^38^, ^51^ ^]^ Each graphene film carried a thin PMMA support layer. After transfer, the PMMA/graphene/gold chips were air‐dried for 30 min, cured at 150 °C for 1 h, and stored under vacuum overnight at room temperature to ensure full contact between graphene and the gold surface. The PMMA was then removed by immersion in acetone (1 h, 50 °C), followed by isopropanol (1 h, room temperature). Finally, the chips were rinsed with acetone and dried under nitrogen flow. Atomic force microscopy (AFM), Raman spectroscopy and scanning electron microscopy (SEM) were performed to validate quality and structural stability of transferred graphene layer, with the data published in the Supporting information of Hasnain et al.^[^ ^12^ ^]^

### Graphene Field‐Effect Transistor (gFET) Fabrication and Electrical Measurements

gFET experiments were performed using flexible, disposable field‐effect transistors (AUFET30, Metrohm) equipped with interdigitated gold finger electrodes on plastic substrates. Monolayer graphene sheets were transferred onto the gold electrodes using the wet transfer method described above, with the curing step modified to 50 °C for 1 h (instead of 150 °C) to prevent melting of the plastic substrate.

Prior to experiments, gFET chips were cleaned with isopropanol, dried under nitrogen, and incubated in 1‐pyrenebutyric acid for 60 min to introduce carboxyl groups. Functionalization steps (NHS/EDC activation, peptide immobilization, ethanolamine blocking) were also identical to those used for graphene‐enhanced SPR chips. Electrical measurements were performed in a liquid‐gated configuration using Ag/AgCl as the gate electrode and C1 buffer as electrolyte. Drain‐source current (I_DS_) was recorded as a function of gate voltage (V_G_) to obtain transfer curves (I_DS_‐V_GS_) of the gFET before and after the cell injection. The charge neutrality point (V_CNP_) was defined as the minimum of the transfer curve. For comparison and clarity between conditions, the gate‐source voltage was referenced to the charge neutrality point (CNP) of the baseline (before cell injection) measurement. A shifted gate voltage was defined as (V_GS_* = V_GS_‐V_CNP, baseline_), such that the baseline curve has V_CNP_ = 0 mV. The transfer curves measured after the cell injection were also shifted by the same offset; the apparent CNP position in V_GS_* therefore corresponds to CNP shift ΔV_CNP_ relative to the baseline.

For cell‐binding experiments, HEK293T cells or neutrophils (100–10 000 cells mL^−1^) were introduced into the fluidic chamber at 10 µL min^−1^. After a 10 min, unbound cells were removed by buffer flushing, followed by a second (I_DS_‐V_GS_) sweep. Shifts in V_CNP_ relative to baseline were quantified as (ΔV_CNP_ = ΔV_CNP, with cells_ – ΔV_CNP, baseline, without cells_). Data were analyzed and classification thresholds were applied to V_CNP_ shifts to calculate precision, recall, and F_1_‐scores (see Section S8, Supporting Information).

### Peptide Functionalization Process

Graphene sensors were primed with 1‐pyrenebutyric acid (PBA, 1 mm in anhydrous DMF) for 60 min, rinsed, and N_2_‐dried. In the flow cell (running buffer; 20 µL min^−1^), a baseline was recorded, then surface carboxylates were activated by a 7‐min injection of EDC/NHS (0.4 m/0.1 m, 1:1, in C1 buffer). Ligand peptides (1 mm in C1) were coupled by flow injection, followed by quenching of residual esters with ethanolamine‐HCl (1 m, pH 8.5, 7 min). Chips were equilibrated in running buffer and immediately used for detection by flowing analytes (HEK293T cells or human neutrophils) at constant rate. This process was followed for both SPR and gFET sensors.

### Statistical Analysis

Statistical analyses were carried out using BioRender R statistical software (version 4.2.2).

For the analysis presented in Figure 5, data were assessed for normality using the Shapiro‐Wilk test and for equality of variances using Levene's test. A Welch's one‐way ANOVA was conducted to evaluate differences between groups. Post hoc pairwise comparisons were performed using Dunnett T3 multiple comparisons test. Each measurement used a single‐channel chip; Target and Control were recorded on separate, nominally identical chips (unpaired comparison). When replicates are reported, each replicate corresponds to a distinct chip measured under the same protocol. All tests were two‐sided (α = 0.05), and exact p‐values and 95% CIs were reported for mean differences.

For the analysis presented in Figure 4 data were assessed for normality using the Shapiro‐Wilk test and for equality of variances using Levene's test. Group differences were assessed using one‐way ANOVA followed by Tukey's multiple comparisons test.

### Isolation of Primary Human Neutrophils

This study used fresh whole blood from healthy adult volunteers purchased from the UK‐based company Research Donors, which provides tested blood for research. Human neutrophils were isolated from this whole blood using the STEMCELL Direct Human Neutrophil Isolation Kit according to manufacturer's protocol. The cells were isolated just prior to the experimental procedures and were stored on ice until their use. The sample size (n) for each collected dataset with neutrophils represents the number of donors, hence neutrophils of each donor account for one biological repetition. In biosensor experiments for each biological repetition two technical repetitions were recorded. Research Donors chose its blood donors randomly and without the knowledge of the experimenter. All experiments were performed according to the relevant guidelines. The influence of sex, gender, or both on the results of the study has not been evaluated.

### HEK293T Cell Culture

HEK293T cells were cultivated in Dulbecco's Modified Eagle's medium supplemented with 10% (v/v) heat‐inactivated fetal calf serum, 1 unit mL^−1^ penicillin‐streptomycin and 2 mm L‐glutamine and were consistently incubated at 37 °C and 5% CO2. HEK293T cells were passaged two times per week and kept below 90% confluence.

### HEK293T Transfection

HEK293T cells were transfected in two different ways depending on their intended assay: a) Standard transient transfection; and b) Nucleofection.

*(a)Transient transfection using jet PEI for calcium imaging or binding studies*: HEK293T cells were seeded to a confluence of 20–30% on black optical 96‐well microplates that were coated with 10 µg mL^−1^ poly‐D‐lysine in Dulbecco's Phosphate Buffered Saline. HEK293T cells were transiently transfected using jetPEI transfection reagent according to manufacturer's protocol once they reached a confluence of 50–70% one day after seeding. For the transfection, 0.125 µg of receptor coding pcDNA3.1 plasmid and 0.125 µg of a G‐protein subunit Gα16 encoding plasmid were added to each well. Receptor plasmids were substituted by an empty pcDNA3.1 vector for Mock negative control. The supernatant of transfected HEK293T cells was replaced by fresh cell culture medium 24 h after transfection and experiments were performed 48 h after transfection.

*(b) Nucleofection for the preparation of HEK293T cells determined for biosensor application*: For biosensor application, nucleofection was performed instead of standard transient transfection because for these assays especially high and uniform transfection rates were crucial due to the low total number of analyzed cells. This was achieved by Lonza nucleofector transfection 2b device using the cell line nucleofector kit V according to the manufacturers protocol on HEK293T cells. For that purpose, equal amounts of FPR2 coding pcDNA3.1 plasmid and GFP‐encoding plasmid were added. FPR2 plasmids were substituted by an empty pcDNA3.1 vector for Mock negative control. GFP was used as a reporter gen, and its expression was continuously monitored for every cell preparation to ensure sufficiently high and uniform transfection rates. After nucleofection, cells were cultivated in 6‐well plates and were harvested 1 day or, at the latest, 2 days after nucleofection.

### Preparation of Cells for Biosensor Application

For biosensor experiments in SPR or electric setup cell suspensions of either nucleofected HEK293T cells or primary human neutrophils were adjusted to a specific cell number. For nucleofected HEK293T this was 100 cells mL^−1^, while for human neutrophils this was 10 000 cells mL^−1^. This was achieved by centrifugation of the isolated or cell culture harvested cells (neutrophils 300 x g for 10 min; HEK293T 1000 x rpm for 3 min) and resuspension in physiological C1 assay buffer (130 mm NaCl, 10 mm HEPES, 5 mm KCl, 2 mm CaCl2, 5 mm glucose, pH 7.4). One control condition in biosensor applications was to block the surface FPR2 receptors. This was achieved by pre‐incubation of cells with 3 µm of Target‐Pep (W‐peptide; WKYMVm‐NH_2_) for 15 min at room temperature. After incubation, cells were washed by two repetition cycles of centrifugation and subsequent resuspension in C1 buffer which was supposed to remove all residual Target‐Pep from cells. For neutrophils, the untreated batch of cells was always prepared similarly in parallel, however, without the addition of Peptide ligand. This enabled a direct comparison between the blocked and the untreated cells by ruling out any effects which were to occur through the extensive cell preparation.

### Calcium Imaging

Calcium Imaging was used to measure cell activation and was performed by using a fluorescence imaging plate reader system (FLIPR tetra or FlexStation III). Transiently transfected HEK293T cells were prepared as described above; isolated human neutrophils were centrifuged at 300 x g for 10 min, resuspended in pre‐mixed Ringer solution (90 mm NaCl, 5 mm HEPES, 2 mm KCl, 2 mm CaCl2, 1 mm MgCl2, 5 mm glucose), calcium sensitive dye was added and neutrophil suspension was subsequently transferred in PDL‐coated black optical 96‐half‐well microplates to a cell density of 2.5 × 10^5^/well. The calcium‐sensitive dye that was used for this assay was Calbryte 520 AM (2 µm), which was prepared in the respective cell buffer (C1 buffer for HEK293T; Ringer solution for neutrophils) and incubated at room temperature. HEK293T cells were loaded with this dye for 2 h, neutrophils were loaded for 1 h while settling onto the assay plates. After the incubation time with the calcium‐sensitive dye, cells were rinsed three times using a microplate washer and were left to rest for 10 min before starting the calcium imaging process. Acquisition of baseline fluorescence (F_0_) was performed for 25 s before ligand application. Calcium responses were calculated as dF/F_0_ values (signal amplitude dF divided by mean baseline fluorescence F_0_).

### Binding Assays and Antibody Staining

Binding assays were performed with transiently transfected HEK293T cells prepared as described above and human neutrophils, which were seeded to a cell density of 3*10^5^cells/well in PDL‐coated optical 96‐well microplates. Cells were incubated with fluorescently labeled W‐peptide and Hoechst 33 342 dye (20 µm) in cell culture media (DMEM for HEK293T; RPMI‐1640 for neutrophils) for 30 min at 37 °C and 5% CO_2_. After the incubation, cells were rinsed 10 times in a plate washer with the respective cell buffer (C1 buffer for HEK293T; Ringer solution for neutrophils). Image acquisition was performed using an ImageXpress Micro high‐throughput confocal microscope with acquisition of three sites per well. Binding analysis was performed using a cell scoring module in the built‐in MetaXpress software. Post‐hoc antibody staining for HEK293T cells was achieved by incubation of ligand‐bound HEK293T cells with monoclonal mouse anti‐fluorescein (FITC) – CF 640R Dye IgG antibody (Sigma, Cat #SAB4600169) at 5 µg mL^−1^ for 1 h in the dark at room temperature. After the incubation, the antibody solution was washed off and cells were imaged.

### Ligands

Peptide stocks were acquired via custom peptide synthesis with purities of >95%. Peptides were stored at −20 °C. Approximately 1 h prior to functional experiments, peptides were thawed at room temperature.

### Cloning of Human FPR Genes

FPR genes were amplified from human genomic DNA and cloned into pcDNA3.1(+) vector as previously described.^[^ ^4^, ^28^ ^]^ Plasmids were introduced into competent *E.coli* bacteria, amplified overnight (rotary incubator, 150 rpm; 2xYT broth), prepared using the Promega PureYield Plasmid Midiprep System according to manufacturer's protocol and purified by isopropanol precipitation. Plasmid concentrations were adjusted to 1 µg µl^−1^. Plasmids were stored at −20 °C. The FPR coding sequences are published in the Supporting Information of Bufe et al.^[^ ^4^ ^]^

## Conflict of Interest

The authors declare no conflict of interest.

## Supporting information



Supporting Information

## Data Availability

The data that support the findings of this study are available from the corresponding author upon reasonable request.
