# Supplementary material for: Cross‐Platform Biosensing of Immune Receptors Using Peptide‐Functionalized Graphene
Source: Adv Sci (Weinh). 2025 Dec 12;13(18):e19436. doi: 10.1002/advs.202519436 (PMC13042608; doi:10.1002/advs.202519436)
Supplement: Supplementary file 1 — Supporting Information [file ADVS-13-e19436-s001.docx]

**Supplementary data**

Cross-Platform Biosensing of Immune Receptors Using Peptide-Functionalized Graphene

*Ahmar Hasnain*^1^, Heiko Heilmann*^2^, Annabel Pohl^1^, Celine Weber^2^, Bernd Bufe^2^, Alexey Tarasov^1a^*

^1^Nanoelectronics & Biosensing Lab, Faculty of Computer Sciences and Microsystems Technology, Kaiserslautern University of Applied Sciences, Amerikastr. 1, 66482 Zweibrücken, Germany

^2^Molecular Immunology Lab, Faculty of Computer Sciences and Microsystems Technology, Kaiserslautern University of Applied Sciences, Amerikastr. 1, 66482 Zweibrücken, Germany

*Contributed equally

^a^ Correspondence & Lead contact: Alexey Tarasov ([alexey.tarasov@hs-kl.de](mailto:alexey.tarasov@hs-kl.de))

1. **Electrical biosensor design and setup**

The electrical biosensing assay was performed using graphene field-effect transistors (gFET). Figure S1 presents the gFET design and characterization. Figure S1A illustrates the physical assembly of the in-house-built flow cell system used for fluidic experiments.


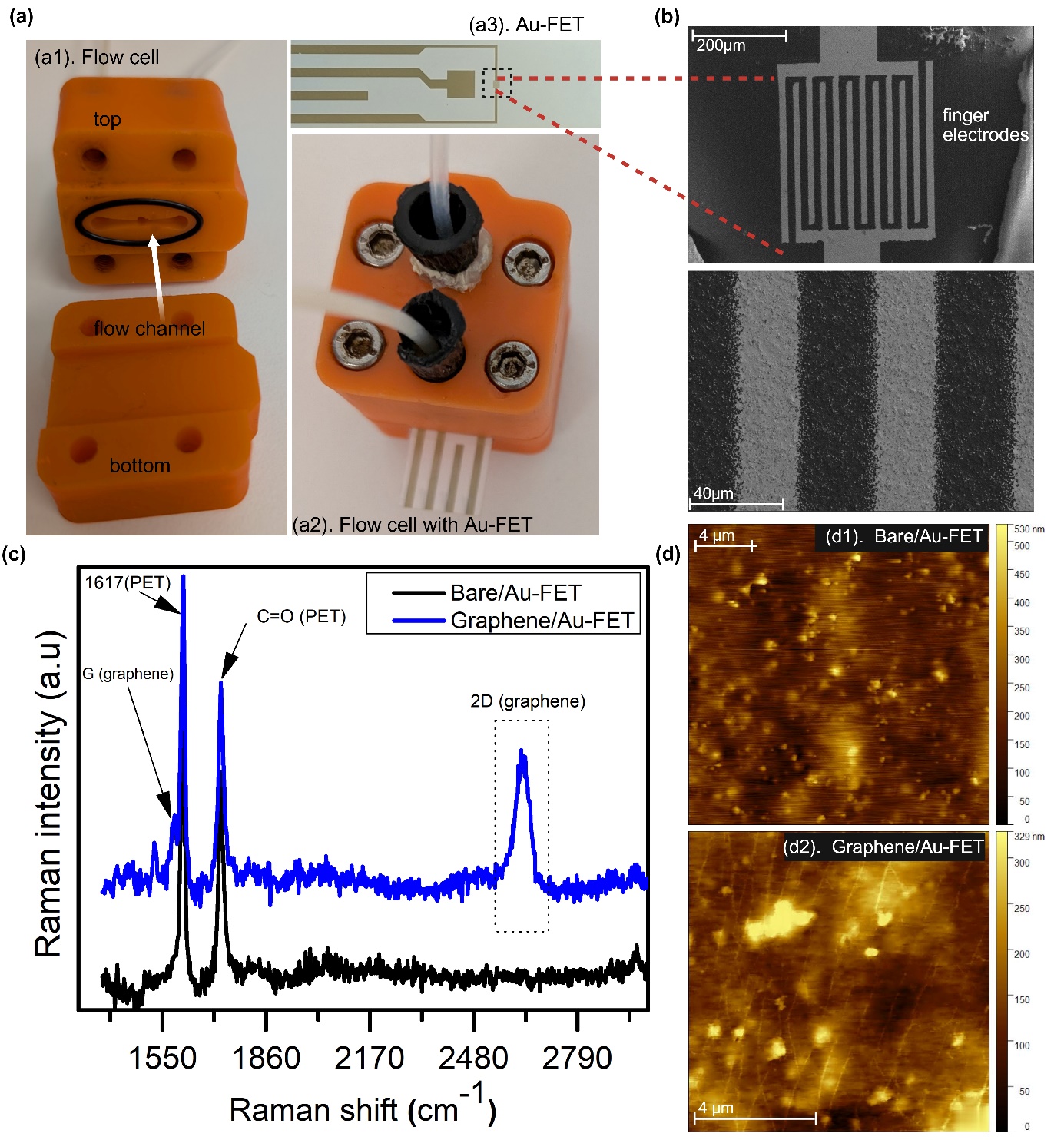


**Figure S1. Characterization and assembly of Au-FET and Graphene/Au-FET devices.** (a) (a1) image of flow cell components showing the top and bottom parts with a flow channel. (a2) Flow cell assembled with Au-FET. (a3) Optical image of the Au-FET device. (b) SEM images of integrated gold finger electrodes (scale bars: 200µm and 40µm). (c) Raman spectra of bare Au-FET(black) and graphene transferred device showing characteristics graphene peaks and PET substrate peaks. (D) AFM images showing surface topography of (d1) bare Au-FET and (d2) Graphene/Au-FET surfaces (Scale bar: 4 µm).

The top and bottom components of the flow cell are closed to sandwich the sensor chip, thereby establishing a flow channel for liquid transmission. Figure S1B shows the interdigitated gold electrodes that serve as source and drain contacts of the FET device, with a gap size of 30 µm. Figure S1C presents Raman spectroscopy data comparing bare Au-FET (black) and graphene-coated Au-FET (blue). The appearance of characteristic graphene peaks, including the G band (~1580 cm^-1^) and 2D band (~2700 cm^-1^), confirms successful transfer of graphene onto the Au-FET. Peaks from the PET substrate are also observed, which appear to overlap with the graphene D peak in the 1345–1355 cm^-1^ region. Figure S1D shows AFM topography images of the bare (d1) and graphene-modified (d2) Au-FET surfaces. The bare PET exhibits a relatively uneven, granular polymer surface with nanoscale texture features. Following graphene transfer, the surface becomes smoother and more uniform, with visible graphene wrinkles and fold lines characteristic of the transferred monolayer.


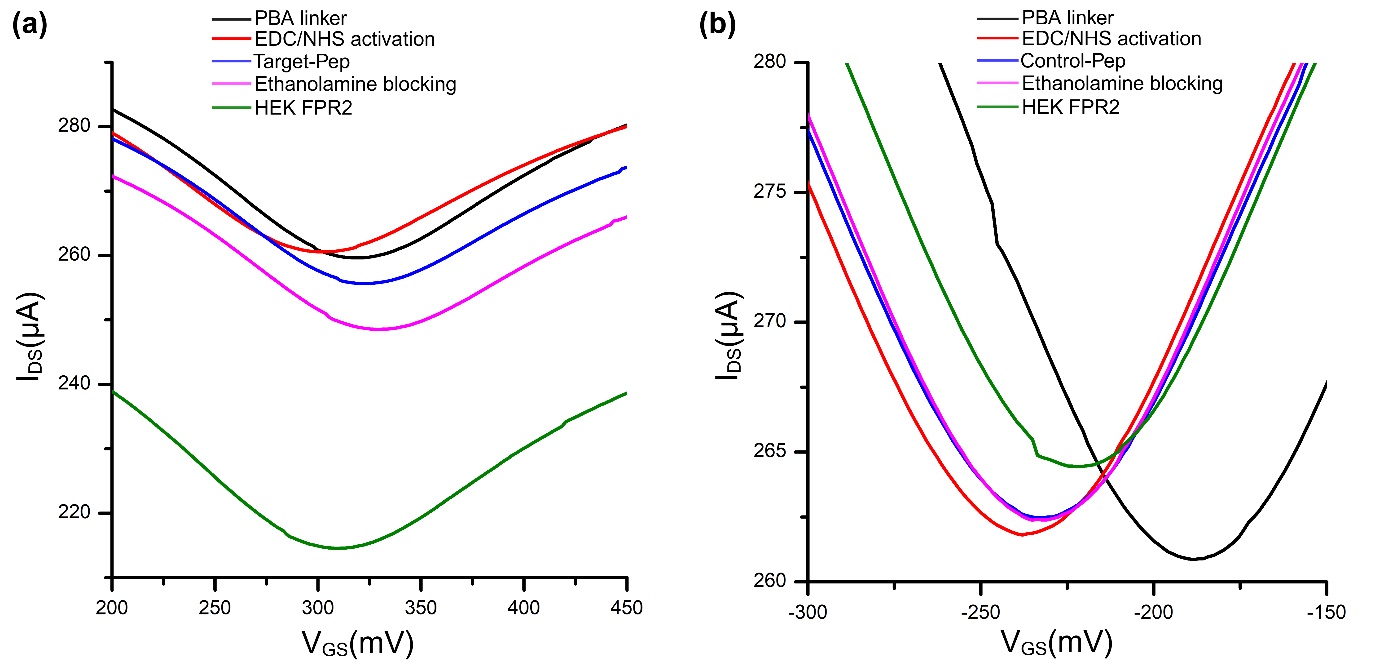


**Figure S2. Electrical biosensor characterization.** (a) Transfer curves (V_GS_-I_DS_) of the gFET biosensor after sequential incubation with the PBA linker (black), EDC/NHS activation (red), Target-Pep immobilization (blue), ethanolamine blocking (magenta), and HEK FPR2 cells (green). (b) Transfer curves obtained upon incubation with the PBA linker (black), EDC/NHS activation (red), Control-Pep immobilization of Control-Pep (blue), ethanolamine blocking (magenta) and HEK FPR2 cells (green).

The transfer curves in **Figure S2**(a-b) show the evolution of the charge neutrality point (V_CNP_) throughout each stage of device functionalization, with I_DS_-V_GS_ curves plotted against the raw gate-source voltage (V_GS_). Following attachment of the PBA linker, the device surface is primed with reactive sites, and subsequent EDC/NHS activation further modifies the interfacial environment. Immobilization of either the target or control peptide produces additional changes in surface charge, evident as shifts in the transfer curves. Ethanolamine treatment then passivates unreacted ester groups. In the final step, the model cell system is introduced: HEK FPR2 cells interact either with Target-Pep (**Figure S2**A) or Control-Pep (**Figure S2**B). For Target-Pep, HEK FPR2 binding induces a pronounced decrease in I_DS_ and a leftwards shift of V_CNP_. In contrast, for Control-Pep, exposure to HEK FPR2 results in a rightward V_CNP_ shift with comparatively modest changes in current. These opposite trends in V_CNP_ evolution underscore the distinct electronic signatures associated with the Target versus Control surface chemistries in this model cell system.

1. **SPR biosensor workflow**

Figure S3 illustrates the workflow of the SPR biosensing assay conducted on graphene-coated sensors. The experiment was carried out in several stages. First, the sensor chip was incubated with linker molecules at ambient temperature. The functionalized chip was then mounted in the SPR chamber, and the measurement sequence was initiated.


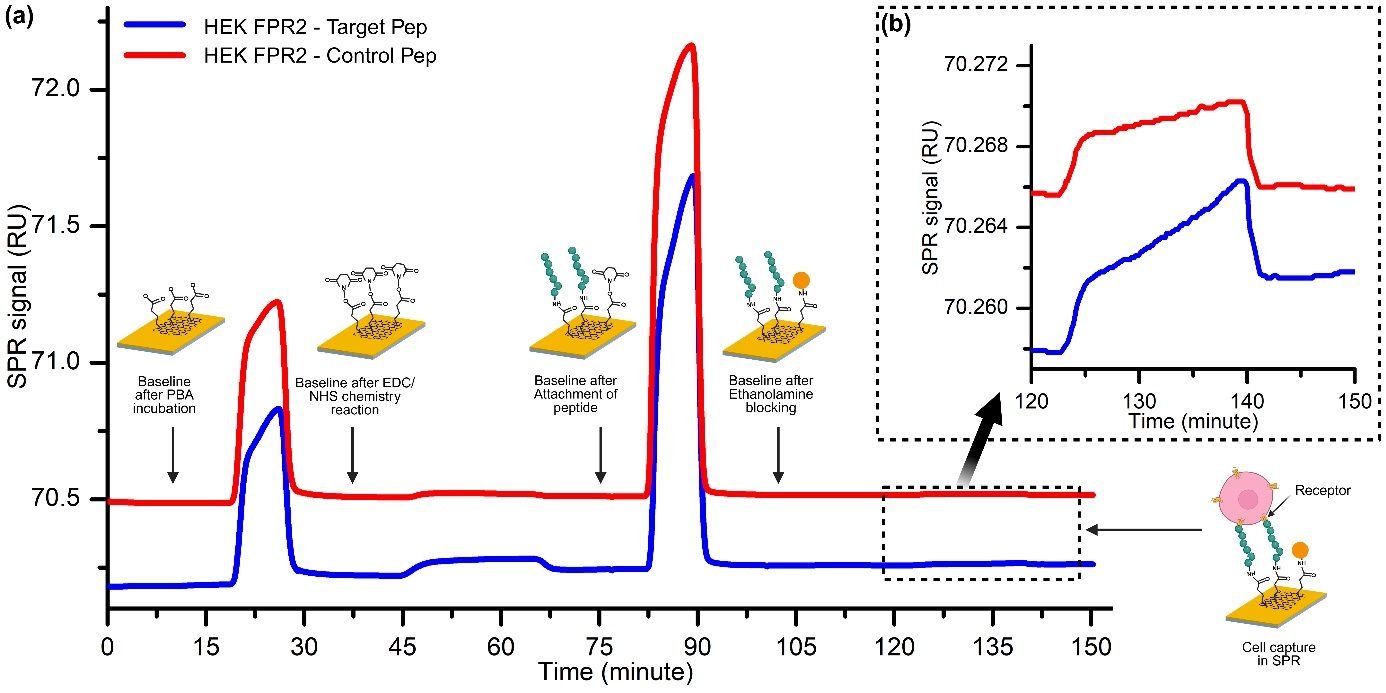


**Figure S3. SPR biosensor** **workflow and cell–peptide interactions.** (a) Real-time SPR sensorgram recorded during the stepwise biosensing assay. Each step of the assay is indicated by an arrow and accompanied by a schematic illustration. (b) Magnified inset highlighting the interaction of HEK293T cells expressing FPR2 with either Target-Pep (blue) or Control-Pep (red).

The sensogram traces represent two independent channels: the blue curve corresponds to the target assay, and the red curve corresponds to the control assay. The procedure was initiated with a running buffer baseline, followed by the injection of EDC/NHS to activate the linker molecules. After a new baseline was established, both target and control peptides were introduced into the respective channels for immobilization. Unreacted esters were then quenched with ethanolamine, restoring the baseline. Subsequently, HEK cells carrying FPR2 receptor were injected. In the target channel, cells interacted with the immobilized target peptide (blue race), while in the control channel cells interacted with the control peptide (red trace) Figure S3B. Each assay step is also illustrated schematically in the Figure S3. The same protocol was applied to study interactions between neutrophils and the target peptide. In this particular instance, neutrophils exhibiting blocked receptors were introduced into the control channel. This approach served as the reference condition, as opposed to utilizing a control peptide.

1. **Target Peptide and Control Peptide**

The Target peptide **WKYMVm-NH_2_** (Figure S4a) is amphipathic, combining hydrophobic residues with basic side chains and net charge of about +1 at physiological pH. The amidated C-terminus blocks the terminal carboxyl, preventing negative charge. Both the N-terminal α-amine and the Lys ε-amine remain available for conjugation. The lowercase m denotes D-Met at the C-terminus. The Control peptide f-MEQQNK (Figure S4b) is hydrophilic, with a net charge of about -1 at physiological pH. The N-terminus is formylated, which blocks its free α-amine and stabilizes the structure. This leaves the Lys ε-amine as the principal nucleophile for conjugation.

On the graphene sensor surface, 1-pyrenebutyric acid (PBA) was first π-π adsorbed to present terminal carboxyl groups, which were subsequently activated with EDC/NHS (Figure S3, Figure S2). Peptides were then covalently immobilized via amide bond formation between the carboxyl groups and the lysine amine. For WKYMVm-NH_2_, coupling can in principle occur at either the α-amine or Lys ε-amine and for f-MEQQNK, N-formylation of the N-terminus inherently directs coupling to Lys ε-amine. To minimize multipoint or non-specific attachment, residual NHS esters were quenched with ethanolamine immediately yielding consistent immobilization levels (see Figure S5a-b).


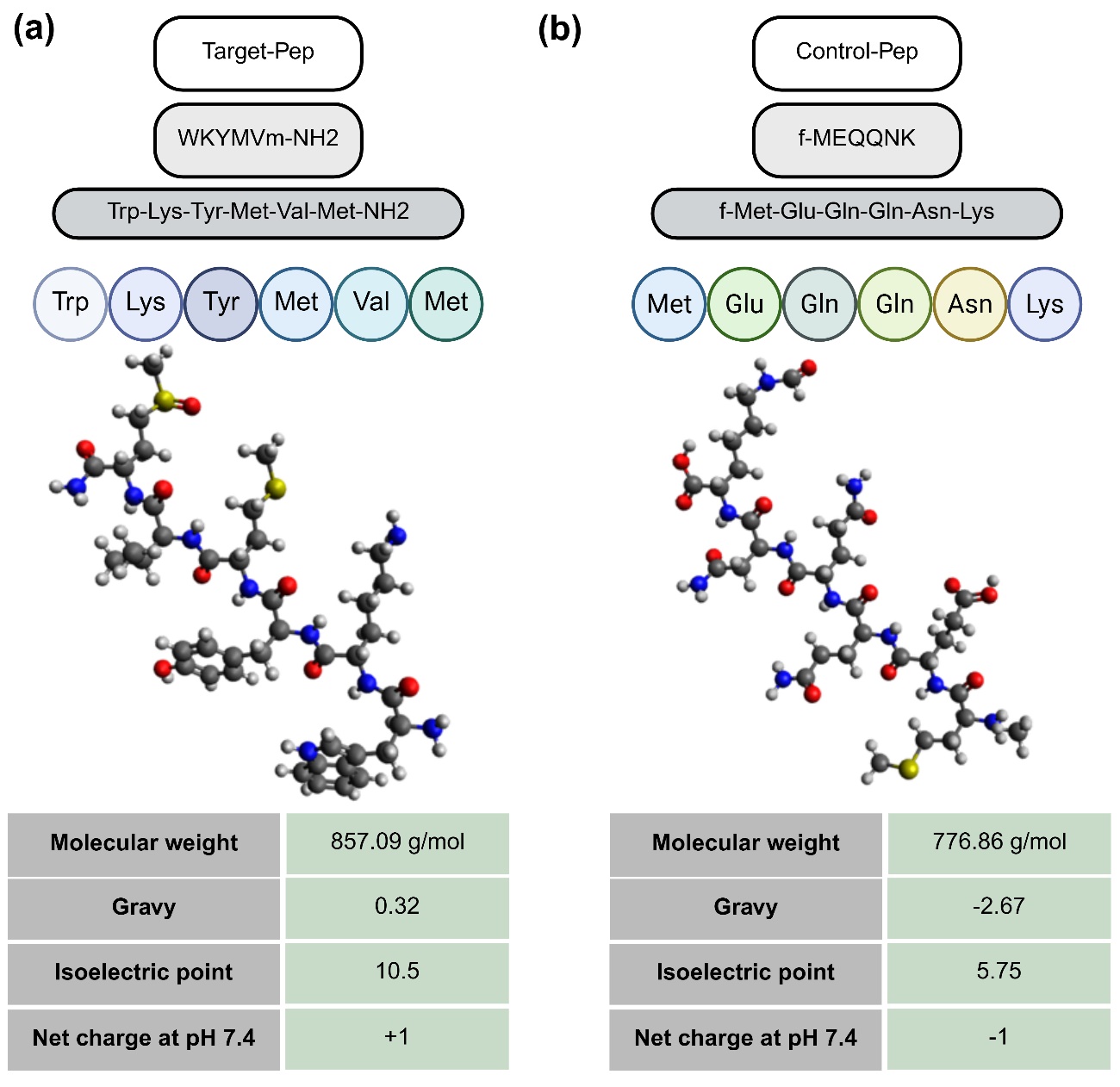


**Figure S4. Structural and physicochemical properties of Target-Pep and Control-Pep.** (a) Amino acid sequence, 3D molecular structure, and physiochemical properties of Target-Pep. (b) Amino acid sequence, 3D molecular structure and physiochemical properties of Control-Pep. Molecular structures were generated using Avogadro.

Real-time immobilization following the activation steps is captured in the SPR sensograms (Figure S3a), and in stepwise gFET response during activation and coupling (Figure S2a-b). The SPR signal in Figure S5a verifies successful peptide immobilization, while the corresponding shift in the charge neutrality point (V_CNP_) in Figure S5b further confirms peptide attachment on the gFET surface. The larger V_CNP_ shift observed for Target-Pep in Figure S5b indicates a more cationic and more densely packed peptide layer, consistent with the increased SPR response in Figure S5a. Reagent concentrations, buffers compositions and incubation timings are provided in Methods section.


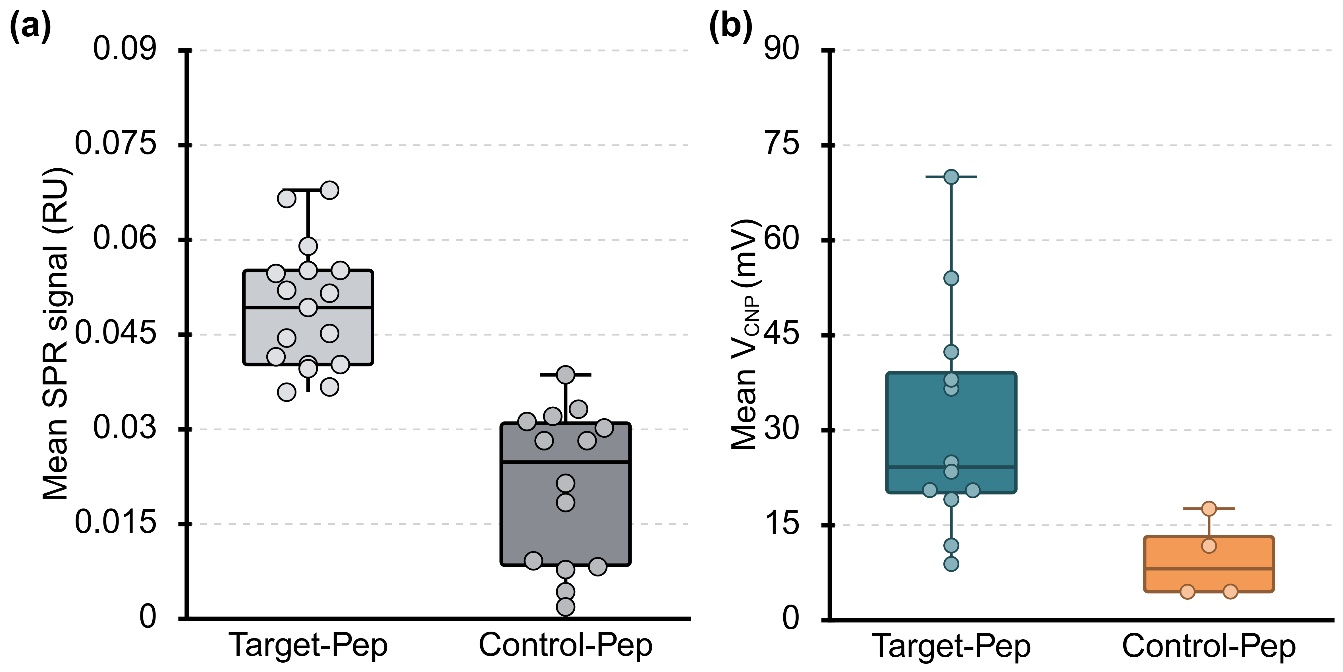


**Figure S5. Immobilization of Target-Pep (WKYMVm-NH₂) and Control-Pep (f-MEQQNK) for the biosensing assay.** (a) Amine-coupling immobilization of Target-Pep and Control-Pep on graphene-coated SPR sensor chips. (b) Amine-coupling immobilization of Target-Pep and Control-Pep on graphene field effect transistors.

1. **Contact angle analysis of electrical biosensing assay**

Contact angle measurements confirmed each stage of surface modification (Figure S6). The bare Au-FET surface exhibited a relatively high contact angle (79.9°), consistent with moderate hydrophobicity. After graphene transfer, the angle decreased slightly to 75.4°, reflecting graphene’s smoother surface and semi-hydrophobic character. Introduction of the PBA linker further lowered the angle to 69.9°, indicating successful deposition of polar functional groups. EDC/NHS activation produced the most hydrophilic surface (60.4°), consistent with the presence of reactive NHS esters. Upon peptide immobilization, the wettability changed according to peptide sequence and orientation. Target-Pep (70.5°), which is positively charged and amphipathic, increased surface hydrophilicity relative to the linker-modified surface. Control-Pep (79.4°), although largely hydrophilic, contains an N-terminal formyl group that limits exposure of polar residues, resulting in a comparatively less hydrophilic surface. Overall, the observed contact angle trends correlate with each peptide’s net charge, hydropathy, and immobilization behavior.


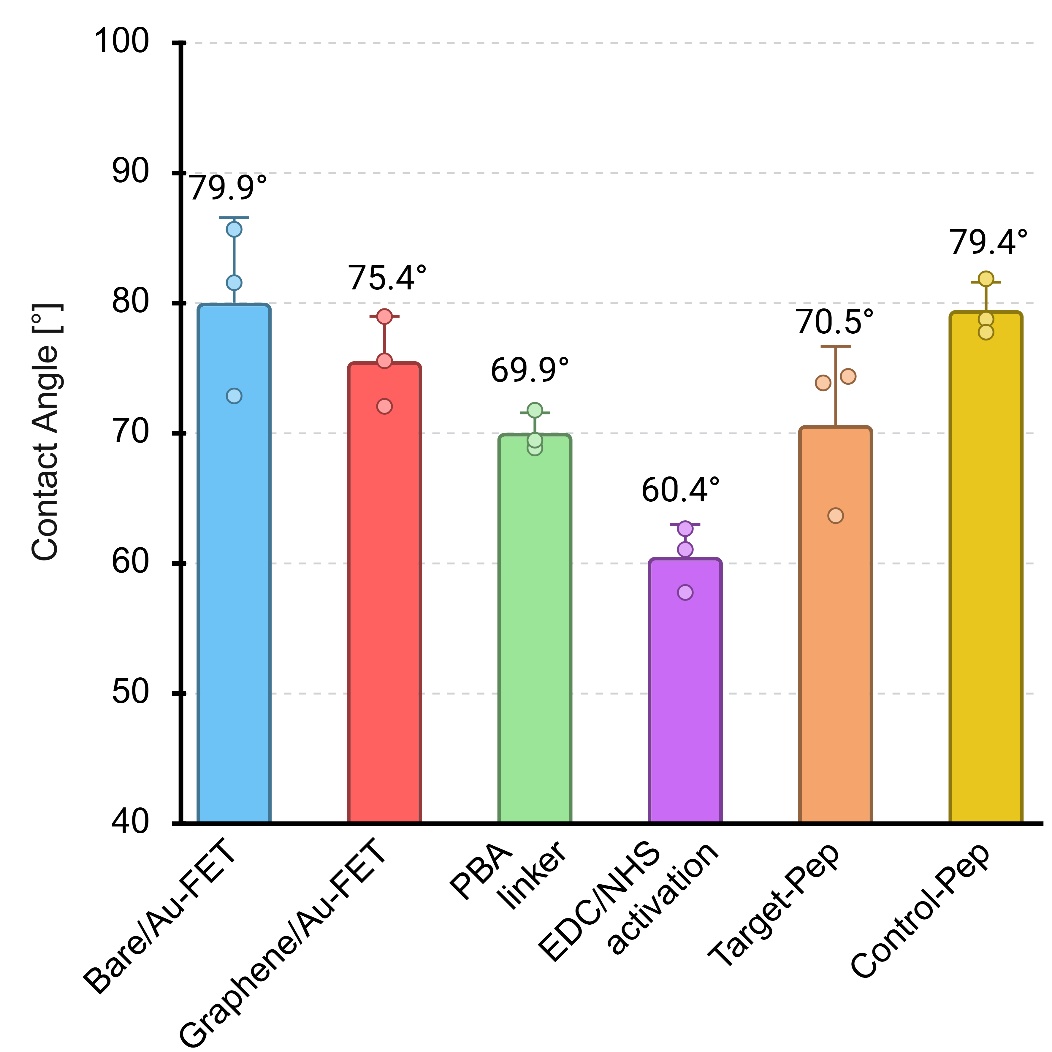


**Figure S6. Water contact angle measurements during stepwise surface functionalization of the electrical biosensor.** The bar graph quantifies contact angles for: bare Au-FET prior to graphene transfer (blue), gFET with monolayer graphene (red), after PBA linker incubation (green), following EDC/NHS activation (purple), Target-Pep immobilization (orange), and Control-Pep immobilization (yellow). Each value represents the average of three measurements. Contact angles were determined using the sessile drop method with a 1 µL droplet.

1. **Calcium flux and binding analysis of receptor-peptide interactions**

To evaluate the interaction of the target peptide (WKYMVm-NH2) and control peptide (f-MEQQNK) with FPRs, we performed calcium imaging on HEK293T cells transfected individually with each FPR subtype. Calcium imaging detects intracellular calcium flux, which reflects peptide–receptor interactions. The target peptide (WKYMVm-NH₂) induced a sensitive, dose-dependent calcium response in both FPR2- (Figure S7a) and FPR1-transfected HEK293T cells (Figure S7c), indicating strong interaction with these receptors. In contrast, the control peptide (f-MEQQNK) did not trigger calcium flux in any of the tested FPRs (Figure S7 b,d). These results demonstrate that the target peptide is suitable for use in the biosensor, while the control peptide serves as an appropriate negative control.


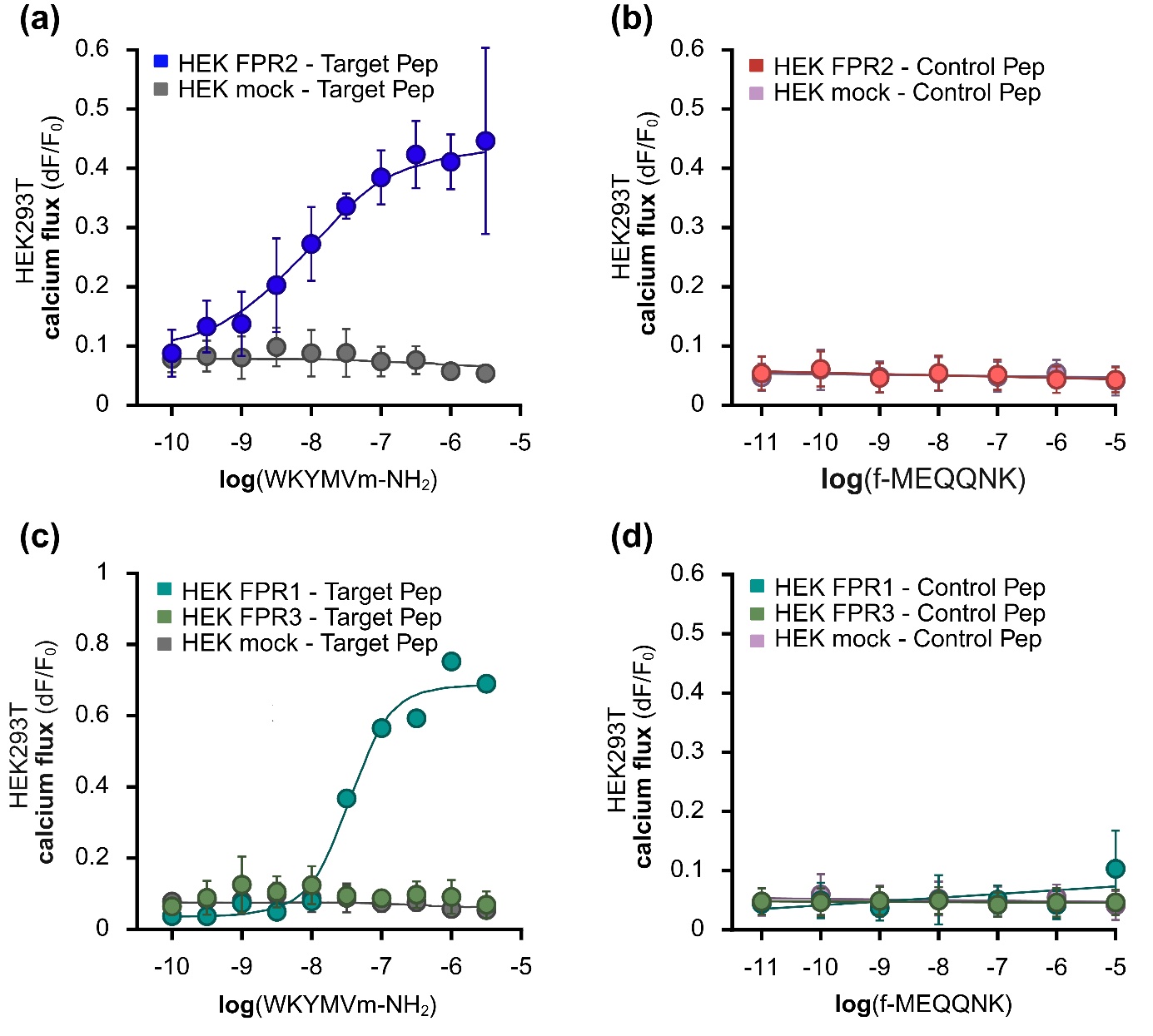


**Figure S7. Target and control peptide-mediated calcium mobilization on FPR1-3.** (a) Mean calcium responses of FPR2- or mock-transfected HEK293T cells to Target-Pep (WKYMVm-NH_2_) (n=3). (b) Mean calcium responses of FPR2- or mock-transfected HEK293T cells to Control-Pep (f-MEQQNK) (n=3). (c) Mean calcium responses of FPR1-, FPR3-, or mock-transfected HEK293T cells to Target-Pep (FPR1 n=1; FPR3 n=3; Mock n=3). (D) Mean calcium responses of FPR1-, FPR3-, or mock-transfected HEK293T cells to Control-Pep (n=3). All data are presented as mean ± SD. Corresponds to main text Figure 3.

Next, we evaluated whether fluorescently labeled Target-Pep (WK(FITC)YMVm-NH₂) could bind to transfected HEK293T cells and whether the FITC tag remained accessible afterward. Accessibility of the FITC tag is critical, as it indicates that surface functionalization, as described in Section 2.2, would be feasible. We focused on FPR2 and FPR1, the two receptors that elicited positive calcium flux in response to Target-Pep. Consistent with the calcium imaging results, both FPR2 and FPR1 (Figure S8a) bound the fluorescently labelled Target-Pep (WK(FITC)YMVm-NH2). However, only FPR2-bound Target-Pep presented an accessible FITC-tag for antibody recognition (Figure 3C), whereas the FITC on FPR1-bound Target-Pep was not accessible (Figure S8b). These findings indicate that peptide-based biosensor interactions are feasible with FPR2 but not with FPR1, guiding the decision to focus on FPR2 for biosensor development.


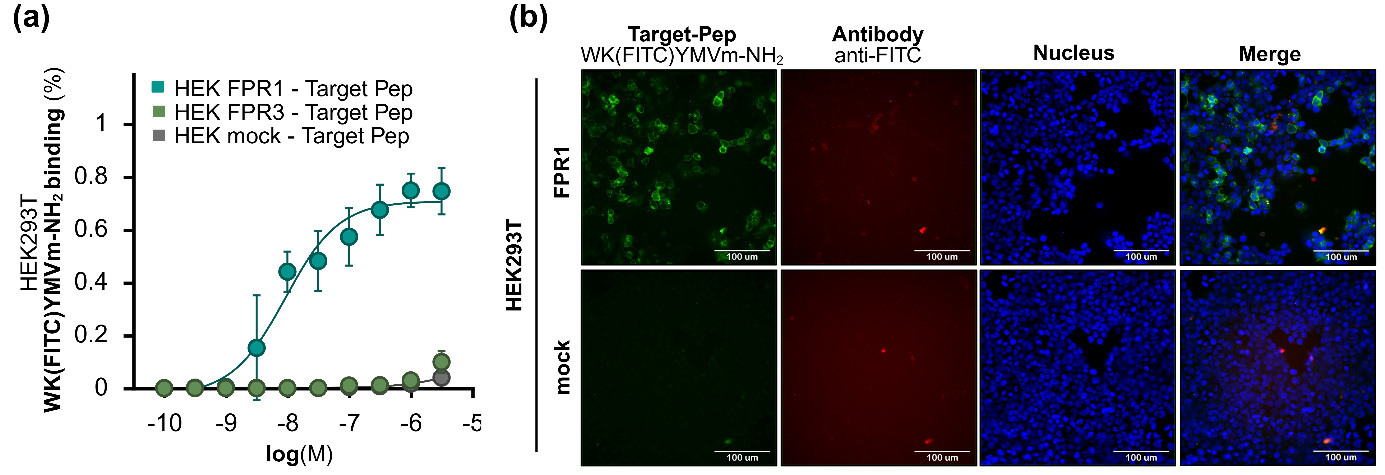


**Figure S8. FPR1 is not a viable receptor target for W-peptide-based biosensing.** (a) Binding kinetics of fluorescently labeled Target-Pep (WK(FITC)YMVm-NH_2_) on FPR1-, FPR3-, or mock-transfected HEK293T cells (n=3). Binding is expressed as the percentage of ligand-bound cells and normalized to the maximum value within each dataset. All data are presented as mean ± SD. (b) Representative confocal images showing binding of FITC-labeled Target peptide WK(FITC)YMVm-NH_2_ (1 µM) binding and subsequent staining with an anti-FITC antibody (5 µg/ml). Nuclei were stained with Hoechst 33342 (20 µM). Scale bar: 100 µm. Corresponds to main text Figure 3.

To extend our studies to primary human cells, we tested the interaction of Target-Pep (WKYMVm-NH₂) with freshly isolated human neutrophils, which naturally express FPR1 and FPR2 on their surface. Calcium imaging revealed a strong response upon peptide treatment, indicating that Target-Pep effectively engages FPRs on these cells (Figure S9). Furthermore, as shown in Figure 6, binding of fluorescently labeled Target-Pep (WK(FITC)YMVm-NH₂) to neutrophil FPRs was also confirmed.


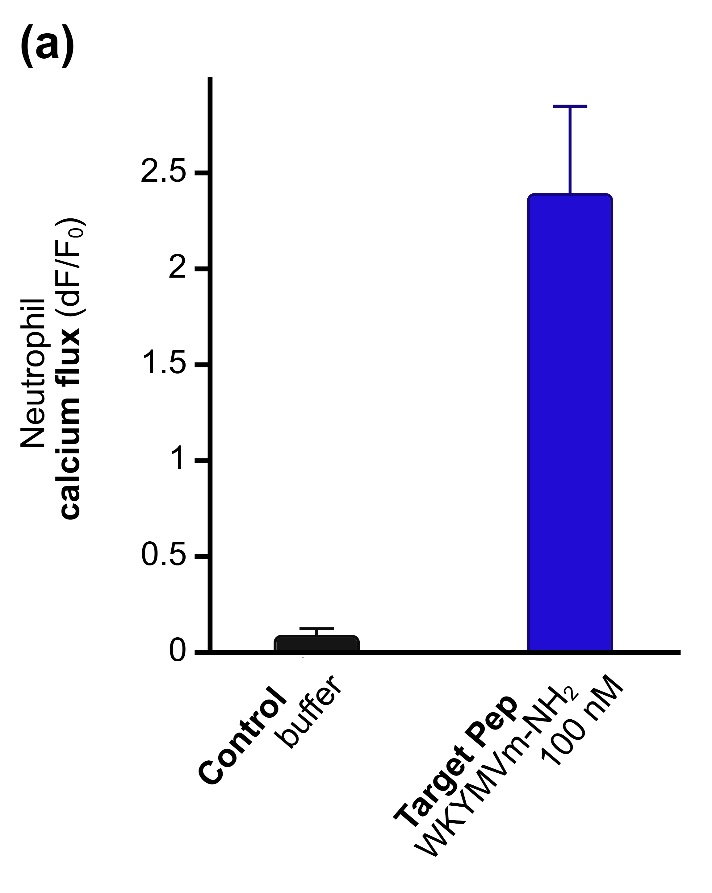


**Figure S9. Target peptide mediated calcium mobilization on human neutrophils.** (a) Mean calcium responses of primarily isolated human neutrophils towards Target-Pep WKYMVm-NH_2_ (100 nM) (n=2). All data are represented as mean +/- SD. Corresponding to main text Figure 6.

1. **Evolution of Drain current at Charge neutrality point in gFET sensor**

In addition to Charge neutrality point data, which is explained in main manuscript, here we also extracted drain current I_DS_ data from (V_GS_-I_DS_) sweeps. The I_DS_ was extracted at Charge neutrality point in the absence and presence of cells, and their difference is plotted in bar graph Figure S10. The blue bars represent the interaction of Target Pep with HEK293T cells transfected with FPR2 receptors (Figure S10a) or human neutrophiles carrying these receptors innately (Figure S10b). The red bars represent the interaction of Control Pep with HEK293T cells transfected with FPR2 receptors (Figure S10a) or human neutrophiles with already blocked receptors with Target Pep (Figure S10b).

The target peptide WKYMVm‑NH₂ produces large negative ΔI_DS_ for receptor‑positive HEK cells and unblocked neutrophils, whereas control peptide with receptor‑positive HEK cells, and target peptide with receptor-blocked neutrophils yield ΔI_DS_≈0 (occasionally small positive values). We interpret negative ΔI_DS_ as the cell footprint becoming less insulating/more electrically permissive at our operating bias, consistent with specific peptide-receptor engagement that reorganizes the membrane-graphene contact (e.g., formation of receptor-rich microcontacts).

Independently, the same peptide elicits receptor‑dependent Ca²⁺ flux in HEK-FPR2 cells and neutrophils (Figure S7a, Figure S8a, Figure S9), verifying that the Target pep can activate its cognate receptor under our assay conditions. While the gFET readout alone does not establish biochemical activation, the receptor‑restricted Ca^2+^ signalling suggests the occurrence of activation‑linked membrane reorganization that reduces the effective insulating gap and enhances interfacial coupling in gFET. We emphasize that this is a plausible contribution rather than a definitive mechanism; alternative factors (adhesion geometry, local ionic environment, peptide charge distribution) may also influence ΔI_DS_.


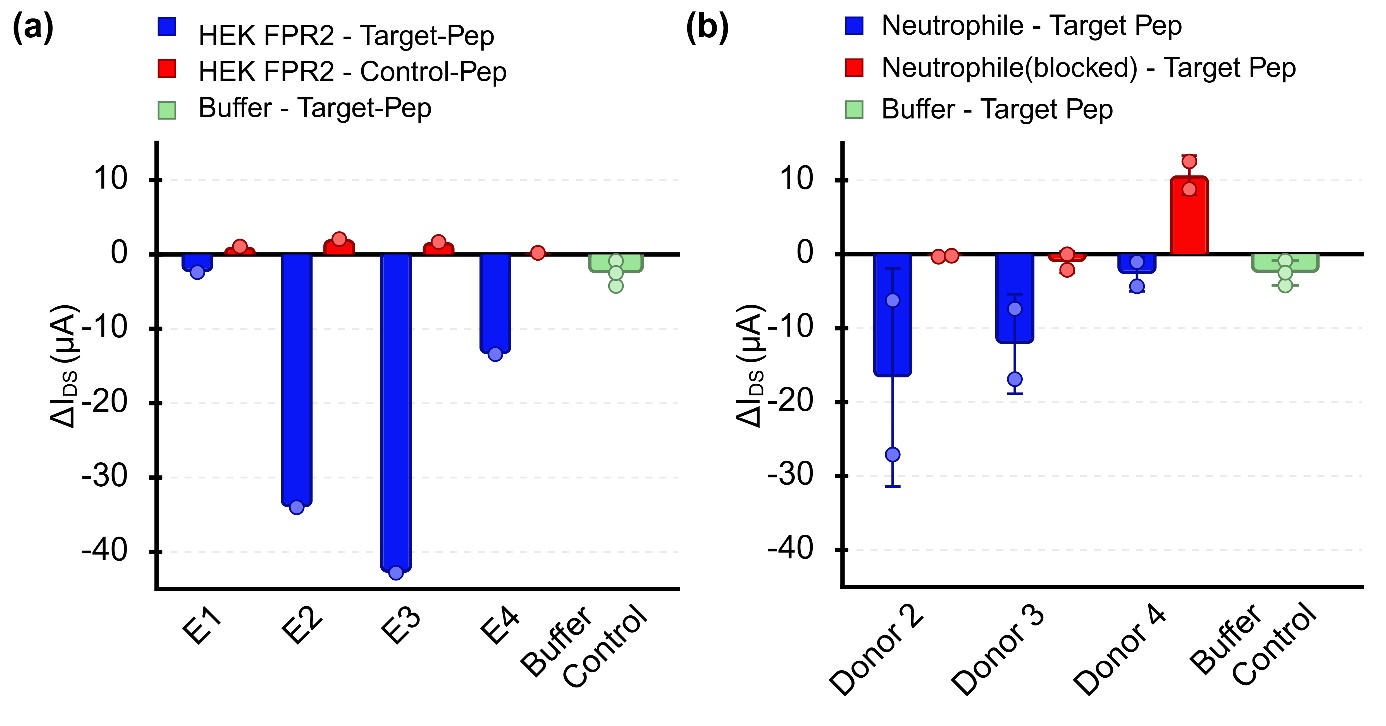


**Figure S10. Analysis of drain current at Charge neutrality point for electrical biosensor.** (a)Quantification of Drain-Source current (I_DS_) shift for interactions of HEK R cells with Target-Pep (WKYMVm-NH₂) and Control-Pep (f-MEQQNK). (b)Quantification of Drain-Source current (I_DS_) shift for interaction of neutrophile and blocked neutrophiles with Target-Pep. Blue bars represent a decrease in I_DS_ and red bars represent an increase due to interaction of cells with immobilized peptides. Green bars represent buffer control.

1. **Optical analysis of FPR2-transfected cells on functionalized slides**

To evaluate the binding specificity of FPR2-expressing cells toward the target peptide WKYMVm-NH_2_, GFP-transfected HEK293T cells were seeded on graphene-coated microscopy slides functionalized with either Target (Figure **S11**b) or the control peptide f-MEQQNK (Figure **S11**C). As an additional negative control, mock-transfected cells were seeded on Target Pep-coated slides (Figure S4D). Following a 30 min incubation to allow for cell attachment, slides underwent three washes with PBS to remove non-adherent cells.


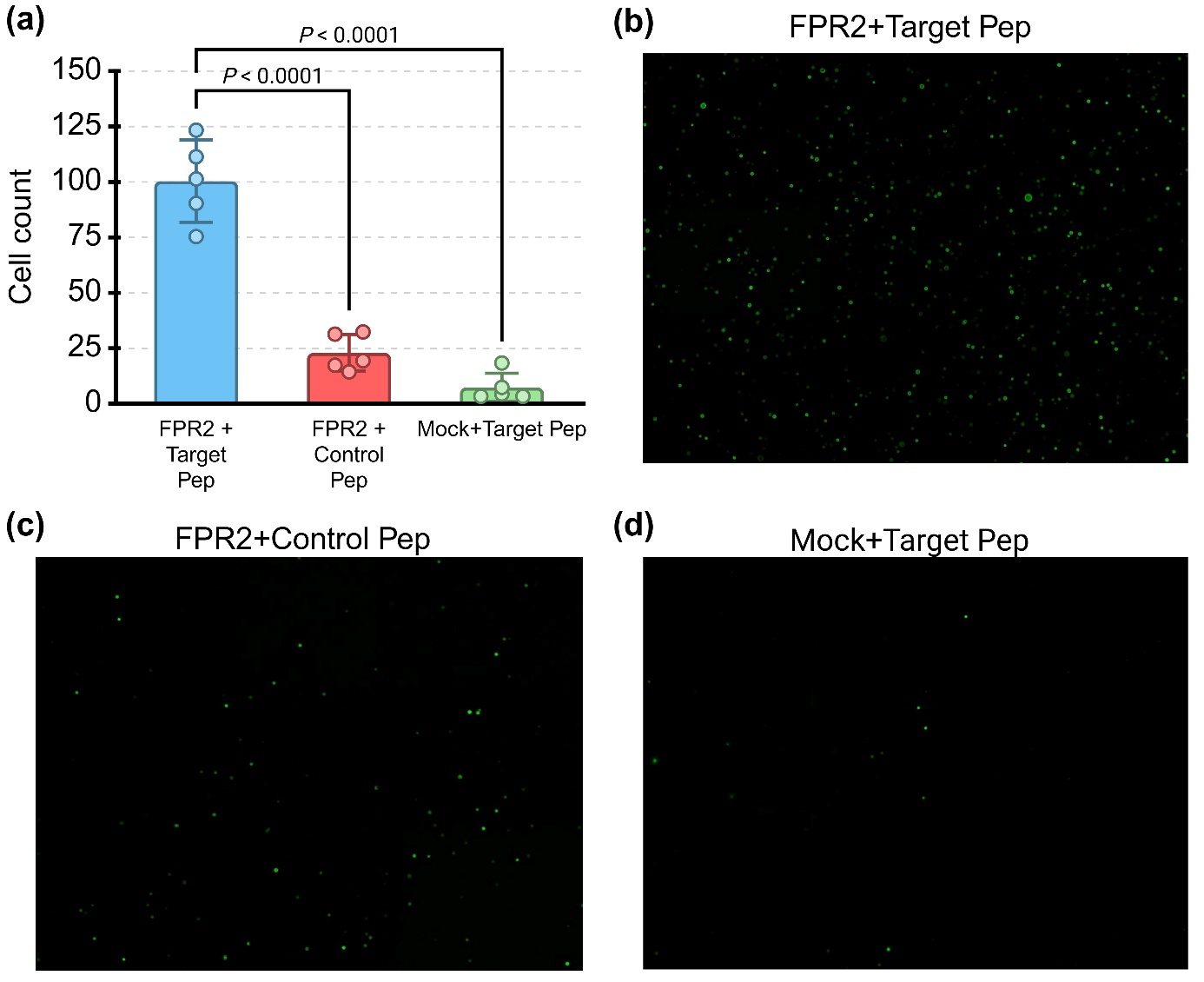


**Figure S11. Adherence of GFP-transfected HEK293T cells on graphene-coated microscopy slides.** (a) Quantification of adhered cells from mosaic images. Cells were counted per condition and per image; individual data points represent counts from each image. Statistical analysis was performed using one-way ANOVA. (b–d) Representative fluorescence images showing GFP-expressing HEK293T cells after 30 min of settling and three subsequent PBS washes: (b) FPR2-transfected cells on Target-Pep–functionalized slides, (c) FPR2-transfected cells on Control-Pep–functionalized slides, and (d) mock-transfected cells on Target-Pep–functionalized slides. Images were acquired using a 395 nm excitation laser and 509 nm emission wavelength. Graphs and analyses were prepared using Biorender.com.

GFP fluorescence was visualized using a 395 nm excitation laser with a 509 nm emission filter, allowing clear detection of bound cells. Quantitative analysis was performed by counting GFP-positive cells per image field (Figure S11a). Target Pep-functionalized slides exhibited a dense and uniform distribution of adherent FPR2-positive cells, while the control peptide resulted in significantly fewer bound cells. Mock-transfected cells on Target Pep-coated slides displayed minimal fluorescence, confirming the specificity of the target peptide-FPR2 interaction. Statistical evaluation by one-way ANOVA revealed highly significant differences (P < 0.0001) between the target condition and both control conditions.

1. **Sensor Classification and F₁ Analysis**

Charge neutrality point V_D_ shifts were measured for target, control, and buffer samples. Leftward ΔV_CNP_ indicates n-type doping; rightward shift indicates p-type doping. The independent unit is a device channel; Target and Control were measured on separate single-channel chips (unpaired). “Target present” if ΔV_CNP_ **≤ threshold** (midpoint between the closest opposite-class values in that cohort); otherwise “Non-Target.” Ties at the threshold were classified as **Target**.

For HEK293T cells transfected with FPR2, Target binding with Target Pep produces leftward (negative) shifts, while Control Pep and buffer produce rightward (positive) or small negative shifts. A threshold of -7.2 mV was selected, positioned between the least negative target (-13.09 mV) and the lowest control (-1.46 mV), to classify positive detections. Using this criterion, all target-induced shifts were correctly identified (TP = 4) with no false positives (FP = 0), and all negatives were correctly excluded (FN = 0, TN = 7). Performance (exact 95% CIs). TP=4, FP=0, FN=0, TN=7 → Precision=1.00, Recall/Sensitivity=1.00, F1=1.00. Exact binomial 95% CIs: Sensitivity 100% (39.8–100%), Specificity 100% (59.0–100%).

Similarly, for neutrophiles, Target binding with Target Pep produced a leftwards (negative) shifts, while Control Pep and buffer produce rightward (positive) shifts. . A threshold of -2.9 mV was selected, positioned between the least negative target (-8.74 mV) and the lowest control (+2.92 mV), to classify positive detections. Using this criterion, all target-induced shifts were correctly identified (TP = 6) with no false positives (FP = 0), and all negatives were correctly excluded (FN = 0, TN = 9).

Performance (exact 95% CIs). TP=6, FP=0, FN=0, TN=9 → Precision=1.00, Recall/Sensitivity=1.00, F1=1.00. Exact binomial 95% CIs: Sensitivity 100% (54.1–100%), Specificity 100% (66.4–100%). Aggregating technical replicates by median per donor, 3/3 donors remained below the threshold (positive).

The dataset is small, and the threshold was chosen pragmatically; future studies with larger sample sets and a broader range of analyte concentrations would allow more robust validation. Threshold sweeps or precision-recall curves could be applied in such datasets to further evaluate sensor performance. Precision, Recall, and F₁-score were calculated using the following formulas:

$\mathbf{Precision=TP/(TP+FP)}$**,** $\mathbf{Recall=TP/(TP+FN)}$**,** $\mathbf{F}_{\mathbf{1}}\mathbf{=}\frac{\boldsymbol{2\times Precision\times Recall}}{\mathbf{Precision+Recall}}$

Notes on limitations: The dataset is small, and the threshold was chosen pragmatically; future studies with larger sample sets and a broader range of analyte concentrations would allow more robust validation. Threshold sweeps or precision-recall curves could be applied in such datasets to further assess sensor performance.
